# The Freshwater Ciliate *Coleps hirtus* as a Model Organism for Metal and Nanoparticle Toxicity: Mixture Interactions and Antioxidant Responses

**DOI:** 10.3390/jox16010023

**Published:** 2026-02-01

**Authors:** Govindhasamay R. Varatharajan, Martina Coletta, Santosh Kumar, Daizy Bharti, Arnab Ghosh, Shikha Singh, Amit C. Kharkwal, Francesco Dondero, Antonietta La Terza

**Affiliations:** 1 School of Biosciences and Veterinary Medicine, University of Camerino, Via Gentile III da Varano, 62032 Camerino, MC, Italy; sarauvraj@gmail.com (G.R.V.); martina.coletta@unicam.it (M.C.); daizybharti83@gmail.com (D.B.); 2 Faculty of Biology, Shenzhen MSU-BIT University, International University Park Road, Dayun New Town, Longgang District, Shenzhen 518172, China; 3 Zoological Survey of India, Prani Vigyan Bhawan, M-Block, New Alipore, Kolkata 700053, India; santoshkumar@zsi.gov.in (S.K.); write2aghosh@gmail.com (A.G.); 4 Amity Institute of Microbial Technology, Amity University Uttar Pradesh (AUUP), Noida 201313, India; shikhasiingh23@gmail.com (S.S.); ackharkwal@amity.edu (A.C.K.); 5 Department of Science and Technological Innovation (DISIT), University of Eastern Piedmont, Viale Teresa Michel 11, 15121 Alessandria, AL, Italy; francesco.dondero@uniupo.it

**Keywords:** cytotoxicity, binary mixture, oxidative stress, reactive oxygen species (ROS), Toxic Unit (TU), MixTOX modeling

## Abstract

Heavy metals (HMs) and metal-oxide nanoparticles (NPs) frequently co-occur in freshwater systems, yet their combined effects on microbial predators remain poorly understood. Here, the freshwater ciliate *Coleps hirtus* was used to evaluate the cytotoxicity of single and binary mixtures of HMs (Cd, Cu, Zn) and NPs (ZnO, CuO, TiO_2_, SiO_2_), and to characterize associated antioxidant responses. Acute toxicity was assessed after 24 h by estimating LC_20_ and LC_50_ values, while mixture toxicity for Cd + Zn and Cd + ZnO was analyzed using the Toxic Unit approach and the MixTOX framework. Non-enzymatic (TPC, DPPH, HRSA) and enzymatic (CAT, GST, GPx, SOD) antioxidants were quantified as sublethal biomarkers at concentrations below lethal thresholds. HMs were markedly more toxic than NPs, with a toxicity ranking of Cu > Cd >> Zn, whereas NPs followed ZnO > CuO >> TiO_2_ >> SiO_2_. Cd + Zn mixtures showed predominantly antagonistic or non-interactive effects, while Cd + ZnO mixtures exhibited strong synergistic toxicity with a non-linear dependence on mixture composition, as supported by MixTox modeling. Exposure to HMs and NPs induced significant and often coordinated changes in antioxidant biomarkers, with binary mixtures eliciting stronger responses than single contaminants. Together, these findings indicate that mixture composition strongly influences both toxicity outcomes and oxidative stress responses in *C. hirtus*. The combination of clear, mixture-dependent toxicity patterns and robust oxidative stress responses makes *C. hirtus* a promising bioindicator for freshwater environments impacted by HMs and NPs.

## 1. Introduction

An ecosystem contains various types of organic and inorganic pollutants, with heavy metals (HMs) and nanoparticles (NPs) being the most prevalent and persistent. HMs such as Cd, Cu, and Zn cannot be degraded and tend to accumulate in sediments and aquatic food webs, originating from both natural processes (e.g., rock weathering, volcanic activity) and numerous human activities, including mining, metallurgy, agriculture, and urban runoff [1,2,3]. In recent years, environmental contamination has become more complex due to the widespread use of engineered nanomaterials [4,5,6]. Metal-based nanoparticles (NPs) (e.g., ZnO, CuO, TiO_2_, SiO_2_), widely incorporated into cosmetics, sunscreens, coatings, textiles, and medical products, enter aquatic systems through wastewater discharge and surface runoff [7,8,9]. Their small size and high reactivity can modify transport, dissolution, and biological uptake compared to bulk metals, potentially altering toxicity profiles. As a result, aquatic organisms are increasingly exposed to mixtures of dissolved metals and NPs, creating new ecotoxicological scenarios that require sensitive model organisms for assessment [10].

Research into the interaction between microorganisms, especially ciliates, and HMs and NPs is currently a trending topic. Ciliates, in particular, play a crucial role in degrading organic matter and/or toxic pollutants in freshwater, soil, and sediments. They are also widely distributed globally and have environmental utility, being used in biomonitoring environmental pollution [11,12] and used as a bioindicator organism [13,14,15,16]. However, studies on the interaction between HMs and NPs in ciliated protozoa are limited compared to available data from other eukaryotic microorganisms such as yeast, fungi, and microalgae. Several ciliate species have been used as test organisms to analyze the cytotoxic effects of HMs and NPs on cellular growth [17,18,19,20,21,22,23,24,25]. Most of these investigations focus on classical model ciliates, whereas species belonging to other taxonomic groups, with distinct morphologies, ecologies, and stress-response strategies, remain insufficiently explored [26,27,28,29].

**Figure 1:**
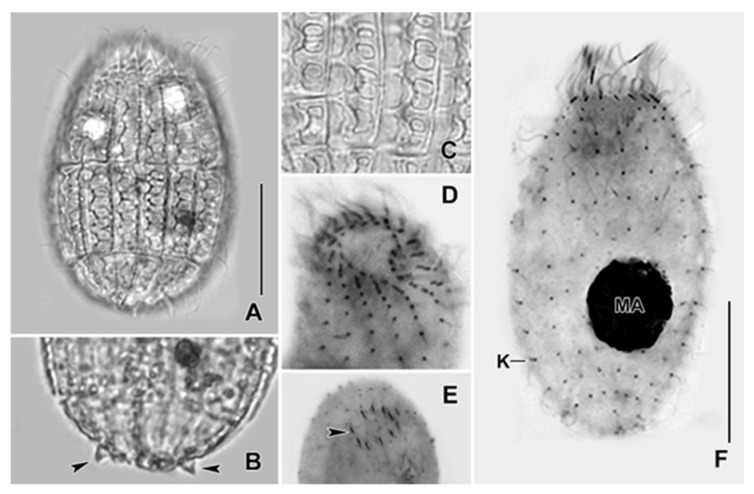
Photomicrographs of *C. hirtus* from life (**A**–**C**) and after protargol impregnation (**D**–**F**). (**A**) Specimen showing the body shape. (**B**) Arrowheads indicate the spines present at the posterior body end. (**C**) Segment showing the shape and arrangement of windows in the calcified plates. (**D**) Segment showing the oral ciliature. (**E**) Presence of extrusomes (arrowhead) in the oral region. (**F**) Specimen showing kinety rows. K, Kinety row; MA, Macronuclear nodule. Scale bars: 20 μm.

In this context, *Coleps hirtus* is of particular interest. *C. hirtus* is cosmopolitan in distribution and a widely studied freshwater ciliate in the protostomatid group, employed for its broad ecological tolerance and feeding flexibility [30,31]. The species measures approximately 50 × 25 µm and possesses a barrel-shaped body armored with calcified plates arranged in 15–16 longitudinal rows, with a single long caudal cilium; the oral aperture lies at the anterior end (Figure 1). It feeds on bacteria, algae, flagellates, other ciliates, and even metazoan tissue (e.g., rotifers, crustaceans, zebrafish), making it both histophagous and scavenging [30,32]. *C. hirtus* may also exhibit cannibalistic behavior, forming coordinated pack-hunting groups [33]. To support its carnivorous feeding, it possesses specialized extrusomes concentrated around the oral region [30,34,35]. Owing to its robust morphology, chemically active extrusomes, and flexible feeding strategies, *C. hirtus* serves as a powerful model organism for studying predator–prey interactions, morphological defenses, and cellular responses to environmental pollutants, including HMs and NPs.

Despite these advantageous biological traits, the ecotoxicological responses of *C. hirtus* have never been characterized. In particular, no data exist regarding its sensitivity to single or combined exposures to HMs and NPs, nor on the antioxidant and cytotoxic biomarker patterns that might support its use as a bioindicator. This gap is notable given the species’ ecological ubiquity and its potential for pollutant uptake through both passive and predatory pathways.

Many studies have shown that metals may stimulate the formation of reactive oxygen species (ROS) in microorganisms [24,36,37]. These ROS can cause damage to biomolecules such as DNA, proteins, and lipids, as well as disrupt redox homeostasis [38], leading to oxidative stress if not controlled by cellular antioxidant defenses. Uncontrolled oxidative stress can result in severe damage to cells, including diseases, aging, and cell death by necrosis and/or apoptosis [39]. Involvement of antioxidant enzymes is an important defense mechanism to protect organisms from oxidative stress. These enzymes include Catalase (CAT), Glutathione S-Transferase (GST), Glutathione Peroxidase (GPx), Superoxide Dismutase (SOD), and non-enzymatic assays such as Total Phenolic Content (TPC), 1, l diphenyl-2-picrylhydrazyl (DPPH) radical scavenging, and Hydroxyl Radical Scavenging Assay (HRSA). Induction of these antioxidant enzymes in response to metals has been reported in many organisms [40,41,42]. Because these biomarkers respond rapidly to environmental stressors, they represent powerful tools for detecting early physiological impairment in aquatic microorganisms.

Ecotoxicological bioassays to detect or evaluate HMs and NPs toxicity have been conducted using freshwater ciliates [19,22,24,31,43,44]. However, none of these studies has examined *C. hirtus*, and it remains unknown whether this species’ unique biology influences its sensitivity to HMs and NPs exposure. Determining its cytotoxic and antioxidant responses is therefore essential for evaluating its potential as a bioindicator species for contaminated habitats.

In this study, our main objective was to evaluate the cytotoxic effects of single and binary mixtures of HMs (Cd, Zn, Cu) and NPs (ZnO, CuO, TiO_2_, and SiO_2_) on a representative freshwater ciliate species, *C. hirtus*, and to characterize its antioxidant responses under these stressors. We aimed to demonstrate the suitability of this species as a bioindicator of habitats polluted by HMs and NPs and to evaluate its antioxidant responses as an effective biomarker for environmental monitoring. Specifically, we quantified 24 h LC_20_ and LC_50_ values, analyzed binary mixture interactions using the Toxic Unit (TU) and MixTOX frameworks [45], and measured key enzymatic (CAT, GST, GPx, SOD) and non-enzymatic (TPC, DPPH, HRSA) antioxidant responses. By integrating toxicity metrics, mixture effects, and biomarker activation, we also assessed whether *C. hirtus* possesses the sensitivity and mechanistic responsiveness required to serve as a reliable freshwater bioindicator species.

## 2. Materials and Methods

### 2.1. Ciliate Strain and Culture Conditions

The experimental organism used in our study was the freshwater ciliate *C. hirtus* isolated from the Sentino River (Genga, AN, Italy) (Figure 1). *C. hirtus* was cultured in Salt Basal Medium (SMB), composed of 1.5 mM NaCl, 0.05 mM KCl, 0.4 mM CaCl_2_, 0.05 mM MgCl_2_, 0.05 mM MgSO_4_, and 2.0 mM phosphate buffer (pH 6.8) [46]. Cultures were maintained at 18 ± 1 °C in the dark and were fed with green algae *Chlorogonium elongatum*. The algae *C. elongatum* was grown in Jaworski’s Medium (JM) [47] at the temperature of 18 ± 1 °C. The JM media was prepared from stock solutions as follows (per 200 mL stock): Ca(NO_3_)_2_·4H_2_O (4.0 g), KH_2_PO_4_ (2.48 g), MgSO_4_·7H_2_O (10.0 g), NaHCO_3_ (3.18 g), Fe–EDTA (EDTA FeNa 0.45 g and EDTA-Na_2_ 0.45 g), H_3_BO_3_ (0.496 g), MnCl_2_·4H_2_O (0.278 g), (NH_4_)_6_Mo_7_O_24_·4H_2_O (0.20 g), cyanocobalamin (0.008 g), thiamine HCl (0.008 g), biotin (0.008 g), NaNO_3_ (16.0 g), and Na_2_HPO_4_·12H_2_O (7.2 g). For medium preparation, 1 mL of each stock solution was added to deionized water and made up to 1 L. All assays were conducted using exponentially growing cells to ensure comparable metabolic states across treatments.

### 2.2. Metal Salts and NPs Stock Preparation

For the ecotoxicological assays, analytical grade pure chemicals (purity ≥ 99%) were used as source of metal ions and NPs: cadmium chloride (anhydrous CdCl_2_), zinc sulphate heptahydrate (ZnSO_4_·7H_2_O) and copper(II) sulphate pentahydrate (CuSO_4_·5H_2_O), zinc oxide (ZnO) (nanopowder, nominal particle size < 100 nm, BET surface area 10–25 m^2^ g^−1^, Sigma-Aldrich product no. 544906), titanium(IV) oxide (TiO_2_) (rutile nanopowder, nominal particle size < 100 nm, BET surface area 50 m^2^ g^−1^, 99.5% trace metals basis, Sigma-Aldrich product no. 637262), silicon dioxide (SiO_2_) (nanopowder, particle size 10–20 nm (BET), 99.5% trace metals basis, Sigma-Aldrich product no. 637238), and copper(II) oxide (CuO) (nanopowder, nominal particle size < 50 nm by TEM, Sigma-Aldrich product no. 544868), all purchased from Sigma-Aldrich (Milan, Italy). Nominal physicochemical properties of the nanomaterials were taken from the supplier’s technical specifications and used as reference information for the present study, including particle size for all nanomaterials and BET surface area for those products for which this information was provided by the supplier. Stock solutions of HMs (0.1 M) and NP stock suspensions (1 g L^−1^) were prepared in SMB medium (pH 7). Working solutions were freshly prepared before each experiment at the following nominal concentrations: 0–7 mg Cd L^−1^, 0–4 mg Cu L^−1^, 0–50 mg Zn L^−1^, 10–150 mg ZnO L^−1^, 1–10 mg CuO L^−1^, 500–8000 mg TiO_2_ L^−1^ and 1000–15,000 mg SiO_2_ L^−1^.

NP stock suspensions were vortexed and briefly sonicated (5–10 min, 40 kHz) prior to dilution to minimize aggregation [48]. This dispersion procedure follows commonly adopted practices in ecotoxicological testing of nanomaterials, which prioritize reproducible and operationally defined preparation of NP suspensions rather than full physicochemical characterization in all cases [48,49]. NP suspensions were freshly prepared for each experiment, and qualitative dispersion stability was monitored by visual inspection for sedimentation or aggregation between 0 and 24 h. Quantitative in-medium characterization of NPs (e.g., hydrodynamic size, zeta potential, or dissolved metal release) was not performed and is explicitly addressed as a limitation of the study in the Discussion. As acknowledged in OECD Test Guideline 318 [49], nanoparticle dispersion stability and agglomeration behavior are highly dependent on medium composition and time, and comprehensive physicochemical characterization is often conducted in dedicated dispersion or fate studies rather than in biological toxicity assays.

### 2.3. Single HMs and NPs Toxicity Tests at 24 h

Preliminary range-finding tests with *C. hirtus* were conducted to identify concentration ranges producing 0–100% mortality for each HM and NP. Based on these preliminary tests, 5–7 concentrations were selected for the final acute toxicity tests. Assays were carried out in 3-well depression glass slides (1 mL final volume). For each biological replicate, three replicate wells were used per concentration. All acute toxicity assays were conducted using three independent biological replicates. Mortality was assessed independently in each replicate following the same experimental protocol.

One hundred cells from exponentially growing cultures were transferred into each well containing SMB with the appropriate test concentration. The setup was covered to prevent evaporation and incubated for 24 h in a humid chamber at 18 ± 1 °C in the dark, without feeding. After 24 h exposure, mortality was assessed using a stereomicroscope (20–40× magnification). Cells were considered dead if they were lysed (i.e., if they disappeared, leaving behind debris) or if they were immobile and unresponsive to gentle mechanical stimulation with a micropipette tip. Percentages of mortality were calculated and used to estimate the LC_20_ (concentration causing mortality in 20% of the cells) and LC_50_ (concentration causing mortality in 50% of the cells) values by logit–log regression. Cell viability was further verified using the Trypan Blue (TB) exclusion assay as described by Strober [50]. Briefly, exposed and control cells were incubated with 0.4% TB solution for 3–5 min and examined under a microscope. Cells that excluded the dye were considered viable, whereas those that took up the dye (i.e., TB-positive cells) were classified as non-viable due to loss of membrane integrity.

### 2.4. Binary Mixture Toxicity Tests (Cd + Zn, Cd + ZnO) at 24 h

Binary mixtures were tested for Cd + Zn and Cd + ZnO using the Toxic Units (TUs) according to Sprague [51]. For each toxicant, 1 TU was defined as the 24 h LC_50_ determined in the single-exposure tests. TU values were calculated from experimentally determined 24 h LC_50_ values, where 1 TU corresponds to the LC_50_ concentration of each compound (Cd: 2.75 mg L^−1^; Zn: 20.42 mg L^−1^; ZnO: 356.18 mg L^−1^). Therefore, all TU-based mixture concentrations can be directly expressed as absolute concentrations (mg L^−1^). Mixtures were prepared by combining the two chemicals at fixed TU ratios, and total mixture concentrations of 0.5, 0.75, 1.0, 1.25, 1.5, 1.75, and 2.0 TUs were tested. The total TU of each mixture was calculated as the sum of the TUs contributed by each component. Expected mortality for each TU combination was obtained from the single-metal concentration–response curves by summing the mortalities corresponding to the TU contribution of each metal in the mixture. For each mixture, observed and expected mortalities were compared. Interactions were classified as synergistic when the observed mortality was significantly higher than the expected value, antagonistic when the observed mortality was significantly lower than the expected value, and as no interaction when no significant deviation from the expected mortality was detected (*p* > 0.05). These TU-based interaction patterns were later complemented and refined by MixTOX modeling (Section 2.8). All binary mixture toxicity assays were conducted using three independent biological replicates. Mortality was assessed independently in each replicate following the same experimental protocol.

### 2.5. Sample Preparation for Antioxidant Analyses

For antioxidant and enzyme analyses, *C. hirtus* cells were exposed for 24 h to selected concentrations of single HMs/NPs and binary mixtures. For each treatment, 2000 cells mL^−1^ from logarithmic-phase cultures were transferred into Petri dishes (10 mL volume) containing SMB with the desired HM/NP concentration or mixture. For single exposures, 0.25, 0.5, 0.75, and 1 TU of Cd, Zn, and ZnO were used. For binary mixtures, sublethal combinations (≤50% mortality) were chosen based on acute toxicity data. In total, nine combinations of Cd + Zn (0.25 + 0.25, 0.5 + 0.25, 0.25 + 0.5, 0.75 + 0.25, 0.5 + 0.5, 0.25 + 0.75, 1 + 0.75, 0.5 + 1, 0.75 + 0.75 TUs) and six of Cd + ZnO (0.25 + 0.25, 0.5 + 0.25, 0.25 + 0.5, 0.75 + 0.25, 0.5 + 0.5, 1 + 0.25 TUs) were used for biomarker measurements. After 24 h, cells were separated from the medium by gentle centrifugation (4000 rpm, 10–15 min). Pellets were washed in distilled water (1000 rpm, 2–3 min) and resuspended in 1 mL of 50 mM phosphate buffer (pH 7.0). Cells were homogenized with a Teflon homogenizer for 4–5 min in the same buffer, and the homogenate was centrifuged at 4000 rpm for 30 min. The supernatant containing intracellular components was collected and stored at −20 °C until analysis. All antioxidant and enzyme assays were performed following Ravindran et al. [52]. All the assays were conducted on three independent biological replicates, with each measurement performed in technical triplicate.

### 2.6. Non-Enzymatic Antioxidant Assays

Three non-enzymatic antioxidant assays were applied to evaluate intracellular antioxidant activity in *C. hirtus* exposed to single and binary mixtures of HMs and NPs: TPC, DPPH radical scavenging, and HRSA.

TPC was determined using the Folin–Ciocalteu method [53] with minor modifications. Briefly, 100 µL of cell extract was mixed with 2 mL of sodium bicarbonate solution and incubated for 2 min at 18 ± 2 °C. Subsequently, 100 µL of Folin–Ciocalteu reagent was added, and the samples were incubated in the dark for 30 min at 18 ± 2 °C. Absorbance was measured at λ = 725 nm using the spectrophotometer (FLUOstar Omega, BMG LABTECH, Ortenberg, Germany). Gallic acid (1 mg/mL) was used as the standard to construct calibration curves, and results were expressed as gallic acid equivalents. Culture medium (without cells) was used as a control.

The DPPH scavenging activity was measured following Yıldırım et al. [54] with minor modifications. 1 mM DPPH stock solution was prepared in 95% ethanol. For each assay, 800 μL of DPPH solution was added to 200 μL of sample extract, incubated for 30 min in the dark at room temperature, then centrifuged at 14,000 rpm for 5 min. The absorbance of the supernatant was measured at λ = 517 nm using a spectrophotometer (FLUOstar Omega, BMG LABTECH, Ortenberg, Germany). Ethanol (95%) was used as a control, and Butylated Hydroxy Anisole (BHA) was used as a reference antioxidant. Scavenging activity (%) was calculated using the following formula:

$$DPPH\;scavenging\;(\%)\;=\;\frac{control\;absorbance\;-\;extract\;absorbance}{control\;absorbance}\;\times 100$$

HRSA was assessed using the Fenton reaction-based assay following Kunchandy and Rao [55] and Ravindran et al. [52] with minor modifications. Hydroxyl radicals were generated in a Fe^3+^ ascorbate EDTA H_2_O_2_ system in 20 mM phosphate buffer (pH 7.4) containing 2.8 mM 2-deoxyribose, 100 µM FeCl_3_, 1 mM H_2_O_2_, and 100 µM EDTA. From this mixture, 800 µL were transferred to test tubes, followed by 10 µL of ascorbic acid (10 mM) and 100 µL cell extract. After incubation at 37 °C for 1 h, 1 mL of 2.8% TCA and 1 mL of 1% TBA were added, and samples were heated at 90 °C for 15 min for colour development. After cooling, absorbance was measured at λ = 532 nm against a blank solution. Mannitol was used as the reference scavenger. The percentage of scavenging was calculated using the following formula:

$$Hydroxyl\;radical\;scavenging\;(\%)\;=\;1\;-\;\frac{Sample\;absorbance}{Blank\;absorbance}\;\times 100$$

### 2.7. Antioxidant Enzyme Assays

Enzymatic antioxidant activities (CAT, GST, GPx, and SOD) were measured in the same extracts used for non-enzymatic assays.

The CAT assay was performed according to the protocol described by Beers and Sizer [56], as adapted by Ravindran et al. [52]. The principle of the catalase assay is based on the ability of the catalase enzyme to degrade hydrogen peroxide into water and oxygen, resulting in a decrease in absorbance. The rate of change in absorbance was measured at λ = 240 nm. The reaction mixture consisted of 2.9 mL of substrate solution, i.e., 0.036% H_2_O_2_ and 100 µL of the sample solution. The blank was prepared using a phosphate buffer. The decomposition of hydrogen peroxide (the substrate) was determined by measuring the reduction in absorbance at λ = 240 nm. One unit was defined as the amount of enzyme required to decompose 1 mM of H_2_O_2_ per minute per milligram of protein at pH 7.0 at 25 °C.

The GST activity was measured by monitoring the conjugation of 1-Chloro, 2, 4-Dinitrobenzene (CDNB) with reduced glutathione (GSH). This reaction was quantified by measuring the increase in absorbance at λ = 340 nm. One unit of enzyme was defined as the amount required to conjugate 10 nmol of CDNB with reduced glutathione per minute at 25 °C. The assay was performed according to the procedure described in Mannervik et al. [57]. The assay cocktail was prepared immediately prior to the assay. For each reaction, the assay cocktail consisted of 980 µL of phosphate-buffered saline (PBS), pH 6.5, 10 µL of 100 mM CDNB (dissolved in ethanol), and 10 µL of 100 mM glutathione. For each sample and the blank, 900 µL of assay cocktail was added directly into the cuvette, and 100 µL of PBS buffer was added to the blank to auto-zero the instrument.

GPx activity was measured according to the method described by Ravindran et al. [52], using guaiacol as the substrate. The principle of the assay is based on the decomposition of hydrogen peroxide by peroxidase, with guaiacol acting as a hydrogen donor, resulting in colour development. The rate of colour development was monitored spectrophotometrically at λ = 436 nm. The assay mixture consisted of 2.8 mL of 0.1 M phosphate buffer (pH 7), 50 µL of 0.018 M guaiacol, and 50 µL of substrate (prepared by diluting 0.1 mL of 30% hydrogen peroxide with distilled water to a final volume of 120 mL; the solution was prepared fresh and stored on ice). The assay mixture was pipetted directly into the cuvette, and 100 µL of the sample was added. A blank was prepared without the sample and used for background subtraction. One unit of enzyme activity was defined as the amount of enzyme that catalyzes the conversion of 1 mmol of hydrogen peroxide per minute.

SOD activity was assayed according to standard protocols [58] by monitoring the inhibition of nitroblue tetrazolium (NBT) reduction at λ = 560 nm. An aliquot of 1 mL of the reagent mixture, consisting of 27 mL of 50 mM Potassium phosphate buffer-pH 7.8, 1 mL Nitroblue tetrazolium (NBT), 1.5 mL of Q Methionine, 0.75 mL of Triton x-100, was dispensed into small glass tubes. To each tube, 20 µL of enzyme extract was added, followed by 10 µL of riboflavin and mixed properly. The reaction mixtures were illuminated for 7 min in an aluminum foil-lined box containing two 20 V fluorescent tubes. Control tubes were prepared without enzyme extract, replacing it with 20 µL of buffer. The absorbance was taken at λ = 560 nm. After the initial reading, the reaction mixtures were re-exposed to light for an additional 7 min by placing the tubes in a cylindrical glass container three-quarters filled with water at 25 °C between two 20 V fluorescent tubes. The increase in absorbance due to formazan formation was recorded at λ = 560 nm at 30 s intervals for up to 5 min. The increase in absorbance in the control tube was taken as 100%, and enzyme activity was calculated as a percentage inhibition per minute. One unit of SOD activity was defined as that amount of enzyme causing 50% inhibition of NBT reduction and was expressed as units/mL.

### 2.8. Statistical Analysis and MixTOX Modeling

Statistical analyses were performed using the InfoStat software (v. 2012) (http://www.infostat.com.ar. accessed on 10 January 2026) [59]. Acute toxicity data were used to estimate 24 h LC_20_ and LC_50_ values for single HMs and NPs by logit-log regression, using log-transformed concentrations and logit-transformed mortality proportions. Prior to parametric analyses, the assumptions of normality and homogeneity of variance were explicitly evaluated for all antioxidant endpoints. Normality was assessed using the Shapiro–Wilk test, and homogeneity of variances was evaluated using Levene’s test for both non-enzymatic (TPC, DPPH, HRSA) and enzymatic (CAT, GST, GPx, SOD) antioxidant parameters. As detailed in Supplementary Tables S1–S4, data were approximately normally distributed and variances were homogeneous across all treatment groups and exposure scenarios, including single-compound exposures and binary mixtures. These results justified the use of parametric statistical tests.

Differences among treatments in mortality and antioxidant responses were evaluated by one-way ANOVA followed by Bonferroni post hoc tests when significant effects were detected. Statistical significance was accepted at *p* < 0.05. Pearson correlation coefficients (R) were calculated to determine relationships among antioxidant endpoints.

Mixture toxicity data for Cd + Zn and Cd + ZnO were further analyzed using the MixTOX tool of Jonker et al. [45], implemented in Microsoft^®^ Excel. Observed mixture responses were compared with predictions of Concentration Addition (CA) and Independent Action (IA) reference models, including deviation functions describing synergism/antagonism (S/A), dose-level dependence (DL), and dose-ratio dependence (DR). Model performance was assessed through goodness-of-fit (*R*^2^) and χ^2^ tests.

## 3. Results

### 3.1. Cytotoxicity of Single HM and NP After 24 h of Exposure

To assess the toxic effects of HMs and NPs on the population growth of the ciliate *C. hirtus*, we performed acute 24 h exposure assays with the metals Cu, Cd, Zn and with the NPs ZnO, CuO, TiO_2_, and SiO_2_. The toxicity ranking for HMs at 24 h was Cu > Cd >> Zn, confirming that Cu was the most toxic element, whereas Zn was the least toxic. For NPs, the toxicity ranking at 24 h was ZnO > CuO >> TiO_2_ >> SiO_2_. During single-metal exposures, *C. hirtus* showed higher resistance to Zn than to Cu at 24 h. Regarding NPs, the cells were highly tolerant to SiO_2_, with no mortality observed at the highest tested concentrations, and relatively tolerant to TiO_2_, which is consistent with the much higher LC values for these NPs.

The 24 h LC_20_ and LC_50_ values for HMs and NPs, obtained by binomial logit regression, are summarized in Table 1. For HMs, LC_50_ values were: 1.62 mg L^−1^ for Cu, 2.75 mg L^−1^ for Cd, and 20.42 mg L^−1^ for Zn. For NPs, LC_50_ values were markedly higher: 447.83 mg L^−1^ for CuO, 356.18 mg L^−1^ for ZnO, and 12.87 g L^−1^ for TiO_2_, while SiO_2_ produced no effects up to 60.08 g L^−1^. A similar trend was observed for LC_20_ values, with HMs showing much lower effective concentrations than NPs (Table 1). Overall, the data demonstrate that Cu, Cd, and Zn were markedly more toxic than ZnO, CuO, and TiO_2_, whereas SiO_2_ was essentially non-toxic under the conditions tested. During exposure to either HMs or NPs, *C. hirtus* cells frequently lost their characteristic barrel-shaped morphology, displayed abnormal swimming behavior, and some became rounded and immobile, particularly at higher concentrations. Based on these acute toxicity patterns, ZnO was selected as the representative NP for subsequent mixture and biomarker analyses, because it combined clear, dose-dependent toxicity with a concentration range that allowed the collection of sub-lethal samples. In contrast, CuO did not provide a sufficiently broad sub-lethal window for reliable physiological assays, whereas TiO_2_ and SiO_2_ showed very limited or no toxicity under comparable conditions.

### 3.2. Cytotoxicity of Binary Mixtures (Cd + Zn and Cd + ZnO) After 24 h of Exposure

Building on the acute toxicity patterns described above, binary mixture experiments were carried out for Cd + Zn and Cd + ZnO, using ZnO as the representative NP in combination with Cd. This design allowed a direct comparison between an ionic-metal mixture (Cd + Zn) and a metal–NP mixture (Cd + ZnO). The expected level of cytotoxicity for each mixture was calculated from the single-compound concentration–response curves, following the Toxic Unit (TU) framework, and compared with the observed mortality (Figure S1a–c).

#### 3.2.1. Toxic Unit (TU) Approach

The toxicity of binary mixtures was evaluated for Cd + Zn and Cd + ZnO using the TU concept. For each pair, mixtures were prepared at total concentrations ranging from 0.5 to 2 TU, with the contribution of each toxicant expressed as a fraction of its single-metal LC_50_ (1 TU). The observed and expected mortalities are reported in Table 2 and Table 3.

In Table 2, each binary mixture is presented with its corresponding Toxic Unit (TU) combination and interaction classification. The total TU of the binary mixture was calculated as the sum of the individual TU fractions of the two metal components. Multiple TU combinations (0.5–2 TU), spanning different dose ratios, were tested to capture potential dose- and ratio-dependent interaction patterns. The expected mortality rates were calculated as the sum of the toxic effects predicted from the single-compound concentration–response curves at the corresponding TU fractions. When the observed mortality was significantly higher than the expected value, the interaction was classified as synergistic, whereas significantly lower observed mortality indicated antagonism; cases without significant deviation were considered additive or non-interactive, following established mixture toxicity criteria [60,61,62,63].

In the Cd + Zn mixture at 24 h, interactions were predominantly antagonistic (7 antagonistic combinations), with only 4 synergistic ones and 4 combinations showing no significant difference between observed and expected mortality (Table 2). This pattern indicates that, in most cases, the presence of Zn tended to reduce the toxicity expected from Cd and vice versa. Notably, some low-TU combinations exhibited synergistic responses, whereas higher TU combinations showed antagonistic effects. Such shifts are consistent with dose- and ratio-dependent interaction dynamics rather than experimental inconsistency. This behavior likely reflects differential metal uptake and cellular stress responses, including competition for transport systems and the induction of detoxification or sequestration pathways at higher Zn contributions, which reduce net toxicity. Detailed TU combinations and interaction classification are provided in Table 2. In contrast, the Cd + ZnO mixture at 24 h showed a completely different behavior. Almost all TU combinations exhibited clear synergistic effects with observed mortality exceeding that expected from additivity (Table 3). Only the highest total concentration (2 TU, 1 + 1) showed no significant deviation from expected mortality. Overall, these results indicate a strong and persistent synergistic interaction across a wide range of TU combinations, demonstrating that Cd markedly enhances ZnO toxicity under most tested conditions.

#### 3.2.2. MixTOX Modeling

Binary mixture data were further analyzed with the MixTOX tool [39] using the Concentration Addition (CA) and Independent Action (IA) reference models and their deviation functions (S/A, DL, DR). The corresponding parameters and goodness-of-fit statistics are reported in Table 4 and Table 5.

For the Cd + Zn mixture at 24 h, the IA model provided the best overall description of the data, explaining 93% of the variance (*R*^2^ = 0.93), whereas CA and its deviation models explained between 88% and 91% (Table 4). No IA-based deviation model (S/A, DL, DR) significantly improved the fit compared to IA alone (*p* (*χ^2^*) > 0.05), indicating that Cd and Zn behaved largely as independent toxicants with modest deviations. Among CA-based models, the CA/DL (dose-level-dependent) deviation model performed relatively better and suggested some degree of antagonism at higher dose levels, consistent with the TU analysis.

For the Cd + ZnO mixture at 24 h, both CA- and IA-based basic models performed poorly unless deviation functions were included (Table 5). The CA model alone explained only 77% of the variance, whereas CA/S/A, CA/DL, and especially CA/DR models considerably improved the fit, reaching *R*^2^ values up to 0.96. Similarly, IA alone explained only 33% of the variance, but IA/S/A, IA/DL, and particularly IA/DR reached *R*^2^ values between 0.90 and 0.96. The best-fitting model overall was IA/DR (*R*^2^ = 0.96), indicating strong dose-ratio-dependent synergism between Cd and ZnO.

**Figure 2:**
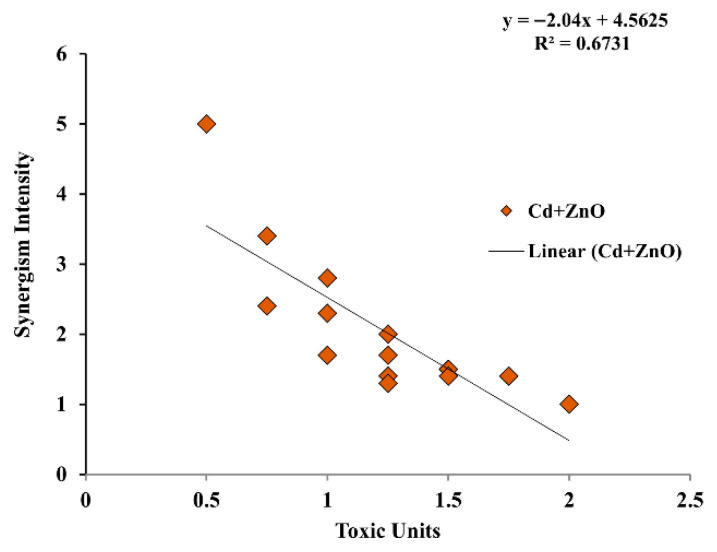
Dose-level-dependent synergism in the Cd + ZnO binary mixture (24 h).

Overall, MixTOX modeling confirms that Cd + Zn mixtures exhibit predominantly antagonistic or near-independent interactions, whereas Cd + ZnO mixtures show persistent synergism across most TU combinations. Importantly, the strength of synergism in the Cd + ZnO mixture varied as a function of both total dose and dose ratio, as illustrated by the dose-ratio-dependent deviation patterns (Figure 2). At the highest total TU combinations, the intensity of synergism decreased but remained clearly detectable across the tested mixture range (Figure 2).

### 3.3. Antioxidant Properties of C. hirtus Cell Extracts

#### 3.3.1. Total Phenolic Content (TPC)

**Figure 3:**
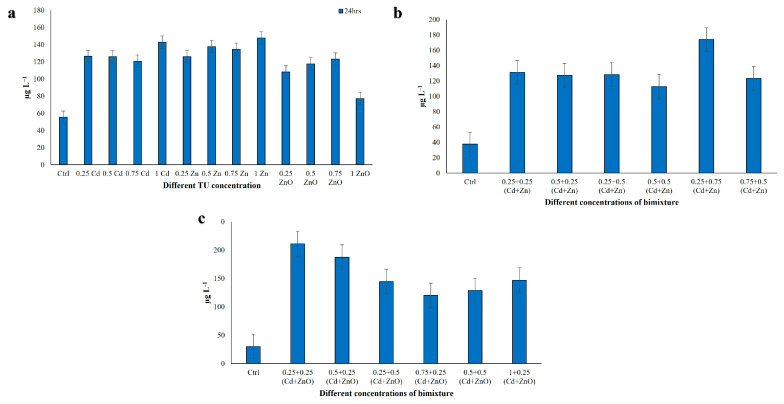
Total phenolic content in *C. hirtus* cell extracts after 24 h exposure to: (**a**) individual HM (Cd, Zn) and NP (ZnO) treatments, (**b**) Cd + Zn mixtures and (**c**) Cd + ZnO mixtures. Results are presented as mean ± SE of three independent biological replicates.

Phenolic compounds such as flavonoids and phenolic acids are known to be significant contributors to the antioxidant capacity of all living organisms [64]. TPC was measured in cell extracts after exposure to single-compound treatments (Cd, Zn, ZnO) and binary mixtures (Cd + Zn and Cd + ZnO) at 24 h (Figure 3). In single-compound exposures, TPC values ranged from 77.26 to 147.71 µg L^−1^ of ciliate extract. The lowest phenolic content was recorded at 1 TU ZnO, while the highest was observed at 1 TU Zn, with all differences being highly significant (*p <* 0.001; Figure 3a).

In the Cd + Zn mixture, TPC ranged between 112.86 and 173.97 µg L^−1^ at 24 h (Figure 3b). The lowest value was found in the 0.5 + 0.5 TU combination, whereas the highest TPC occurred at 0.25 + 0.75 TU (Cd + Zn), again with *p* < 0.001. In the Cd + ZnO mixture, TPC values ranged from 120.06 to 211.15 µg L^−1^ (Figure 3c). Here, the minimum was observed at 0.75 + 0.25 TU (Cd + ZnO), and the maximum at 0.25 + 0.25 TU, with highly significant differences (*p* < 0.001). Overall, TPC was higher in binary mixtures than in single-compound treatments, particularly in Cd + ZnO, suggesting that phenolic metabolites are strongly induced under combined HM-NPs stress. Data met the assumptions for parametric analysis (see Materials and Methods and Supplementary Tables S1 and S2).

#### 3.3.2. DPPH Radical Scavenging Activity

**Figure 4:**
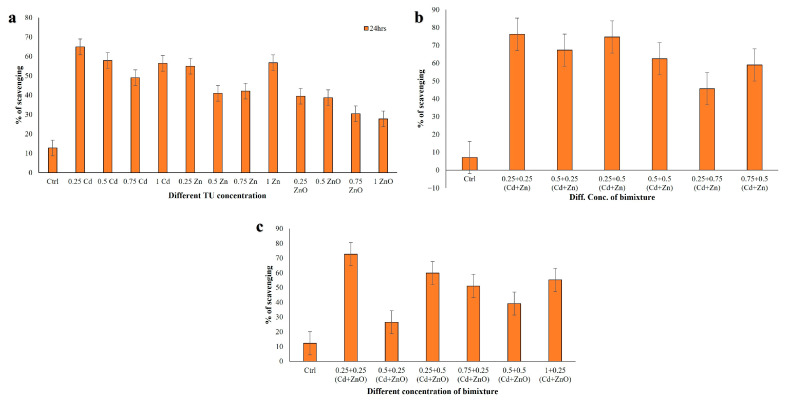
DPPH scavenging activity measured in *C. hirtus* cell extracts after 24 h exposure to: (**a**) individual HM (Cd, Zn) and NP (ZnO) treatments, (**b**) Cd + Zn mixtures and (**c**) Cd + ZnO mixtures. Results are presented as mean ± SE of three independent biological replicates.

DPPH radical scavenging activity was evaluated after 24 h exposure in cell extracts from *C. hirtus* exposed to single-compound treatments (Cd, Zn, ZnO) and to binary mixtures (Cd + Zn and Cd + ZnO) (Figure 4). In single-compound exposures, DPPH scavenging ranged from 27.77% to 64.92% (*p* < 0.001), with the highest activity at 0.25 TU Cd and the lowest at 1 TU ZnO (Figure 4a). In the Cd + Zn mixture, DPPH scavenging activity ranged between 45.71% and 76.22% (Figure 4b). The highest value was observed at 0.25 + 0.25 TU (Cd + Zn), whereas the lowest was recorded at 0.25 + 0.75 TU (Cd + Zn), with all significant at *p* < 0.001. In the Cd + ZnO mixture, DPPH activities varied from 26.51% to 72.69% (Figure 4c). The maximum was at 0.25 + 0.25 TU (Cd + ZnO) and the minimum at 0.5 + 0.25 TU (Cd + ZnO), again with *p* < 0.001. These results indicate that exposure to binary mixtures generally enhanced DPPH scavenging compared to single metals, particularly for low-to-intermediate TU combinations of Cd with either Zn or ZnO. Data met the assumptions for parametric analysis (see Materials and Methods and Supplementary Tables S1 and S2).

#### 3.3.3. Hydroxyl Radical Scavenging Activity (HRSA)

**Figure 5:**
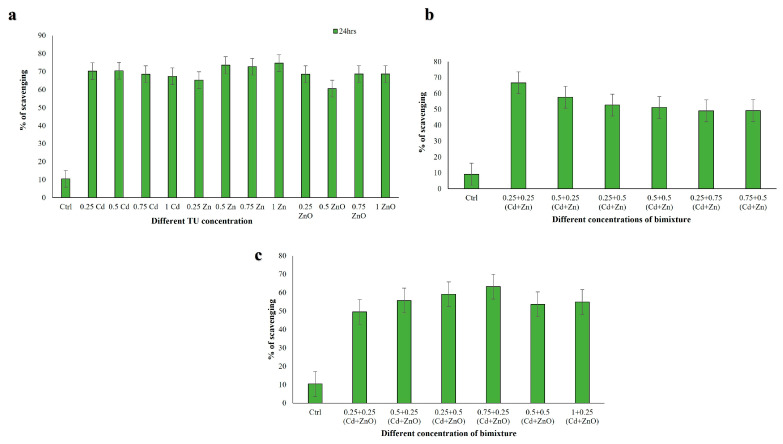
Hydroxyl radical scavenging activity (HRSA) measured in *C. hirtus* cell extracts after 24 h exposure to: (**a**) individual HM (Cd, Zn) and NP (ZnO) treatments, (**b**) Cd + Zn mixtures and (**c**) Cd + ZnO mixtures. Results are presented as mean ± SE of three independent biological replicates.

Hydroxyl radical scavenging activity (HRSA) was also determined in cell extracts after 24 h exposure (Figure 5). The ciliate extracts showed noticeable hydroxyl radical scavenging activity with increasing concentration. In single-compound treatments, HRSA ranged from 60.55% to 74.69%, with the lowest value at 0.5 TU ZnO and the highest at 1 TU Zn (*p* < 0.001; Figure 5a). In Cd + Zn mixtures, HRSA values ranged from 49.10% to 66.66% at 24 h (Figure 5b). The minimum was observed at 0.25 + 0.75 TU (Cd + Zn), with all comparisons significant (*p* < 0.001). In Cd + ZnO mixtures, HRSA ranged from 49.53% to 63.27% (Figure 5c), with the lowest value at 0.25 + 0.25 TU and the highest at 0.5 + 0.25 TU (Cd + ZnO) (*p* < 0.001). In contrast to DPPH and TPC, single-metal treatments (particularly Zn) elicited the highest HRSA values, whereas binary mixtures produced slightly lower but still elevated hydroxyl radical scavenging. This suggests that different antioxidant mechanisms and compounds may be involved in scavenging distinct ROS species. Data met the assumptions for parametric analysis (see Materials and Methods and Supplementary Tables S1 and S2).

### 3.4. Antioxidant Enzyme Activities

#### 3.4.1. Catalase (CAT) Assay

**Figure 6:**
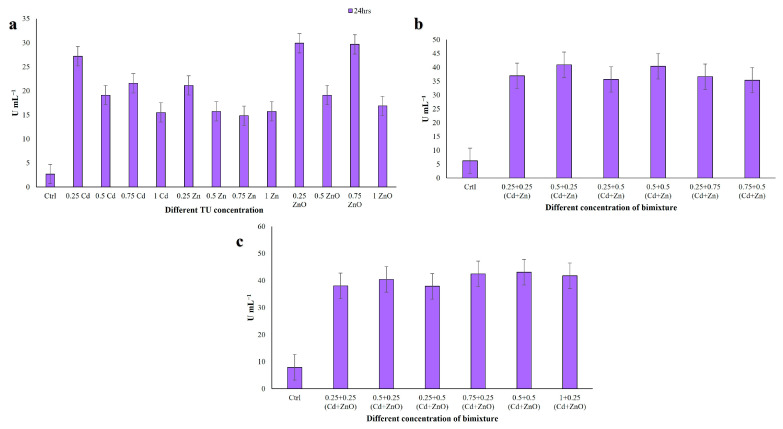
Catalase activity measured in *C. hirtus* cell extracts after 24 h exposure to: (**a**) individual HM (Cd, Zn) and NP (ZnO) treatments, (**b**) Cd + Zn mixtures and (**c**) Cd + ZnO mixtures. Results are presented as mean ± SE of three independent biological replicates.

CAT activity in *C. hirtus* extracts ranged from 14.84 to 29.91 U mL^−1^ after 24 h single-compound exposure (Figure 6a). The lowest activity was detected at 0.25 TU ZnO, whereas the highest was at 0.75 TU Zn. In the Cd + Zn mixture, CAT activity ranged from 35.36 to 40.41 U mL^−1^ (Figure 6b), with the minimum at 0.75 + 0.5 TU and the maximum at 0.5 + 0.5 TU (Cd + Zn). In the Cd + ZnO mixture, CAT activity varied between 37.90 and 43.12 U mL^−1^ (Figure 6c), with the lowest value at 0.25 + 0.5 TU and the highest at 0.5 + 0.5 TU (Cd + ZnO). Thus, CAT activity was consistently higher in binary mixture treatments (Cd + Zn, Cd + ZnO) than in single-compound exposures (Cd, Zn, ZnO), with the Cd + ZnO combination eliciting the strongest response. Data met the assumptions for parametric analysis (see Materials and Methods and Supplementary Tables S3 and S4).

#### 3.4.2. Glutathione S-Transferase (GST) Assay

**Figure 7:**
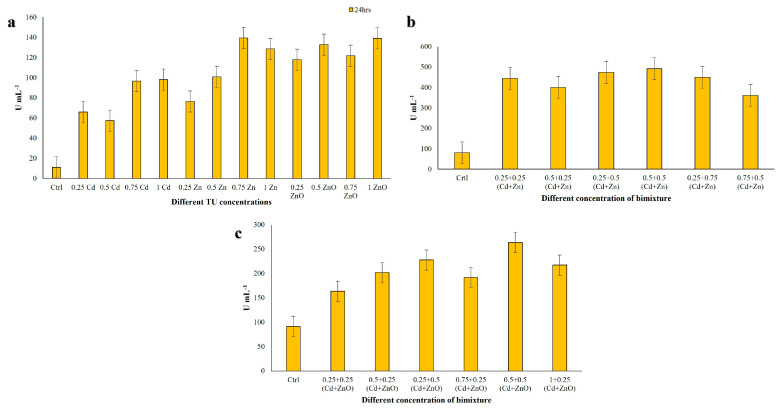
Glutathione S-transferase (GST) activity measured in *C. hirtus* cell extracts after 24 h exposure to: (**a**) individual HM (Cd, Zn) and NP (ZnO) treatments, (**b**) Cd + Zn mixtures and (**c**) Cd + ZnO mixtures. Results are presented as mean ± SE of three independent biological replicates.

GST catalyzes the conjugation of the reduced form of glutathione to xenobiotic substrates for detoxification purposes. GST activity in single-compound treatments ranged from 57.29 to 139.24 U mL^−1^ (Figure 7a). The lowest activity was observed at 0.5 TU Cd and the highest at 0.75 TU Zn. In the Cd + Zn mixture at 24 h, GST activity increased substantially, ranging from 361.11 to 492.03 U mL^−1^ (Figure 7b). The highest level was recorded at 0.5 + 0.5 TU (Cd + Zn) and the lowest at 0.75 + 0.5 TU (Cd + Zn). In the Cd + ZnO mixture, GST activity ranged from 163.54 to 263.54 U mL^−1^ (Figure 7c), with the minimum at 0.25 + 0.25 TU (Cd + ZnO) and the maximum at 0.5 + 0.5 TU (Cd + ZnO). These results indicate a strong induction of GST under co-exposure, especially in Cd + Zn mixtures, suggesting a key role for glutathione-dependent detoxification pathways in response to metal and nanoparticle stress. Data met the assumptions for parametric analysis (see Materials and Methods and Supplementary Tables S3 and S4).

#### 3.4.3. Guaiacol Peroxidase (GPx) Assay

**Figure 8:**
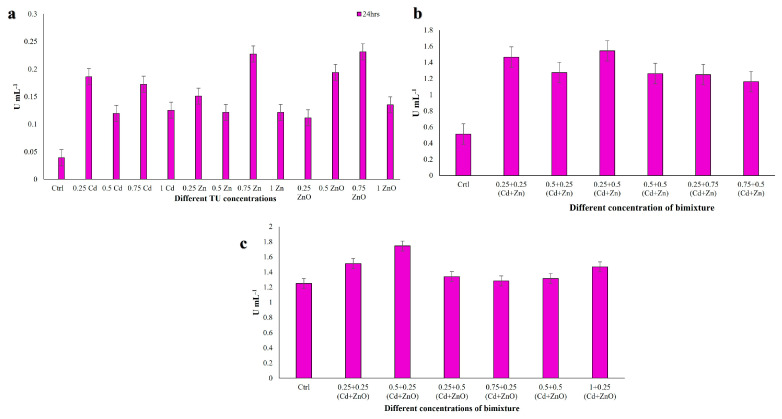
Guaiacol peroxidase (GPx) activity measured in *C. hirtus* cell extracts after 24 h exposure to: (**a**) individual HM (Cd, Zn) and NP (ZnO) treatments, (**b**) Cd + Zn mixtures and (**c**) Cd + ZnO mixtures. Results are presented as mean ± SE of three independent biological replicates.

Peroxidases play a key role in enhancing a cell’s defenses against pathogens and environmental stresses. GPx activity in single-compound treatments ranged between 0.11 and 0.23 U mL^−1^ (Figure 8a). The minimum value was recorded at 0.75 TU ZnO, and the maximum at 0.25 TU ZnO. In Cd + Zn mixtures, GPx activity increased markedly, ranging from 1.16 to 1.54 U mL^−1^ (Figure 8b). The lowest activity was observed at 0.75 + 0.5 TU and the highest at 0.25 + 0.5 TU (Cd + Zn). In Cd + ZnO mixtures, GPx activity further increased, ranging from 1.24 to 1.75 U mL^−1^ (Figure 8c), with a minimum at 0.75 + 0.25 TU and a maximum at 0.5 + 0.25 TU (Cd + ZnO). Overall, GPx activity was considerably higher in binary mixtures than in single-compound treatments, with Cd + ZnO producing the strongest induction, consistent with the synergistic toxicity of this mixture. Data met the assumptions for parametric analysis (see Materials and Methods and Supplementary Tables S3 and S4).

#### 3.4.4. Superoxide Dismutase (SOD) Assay

**Figure 9:**
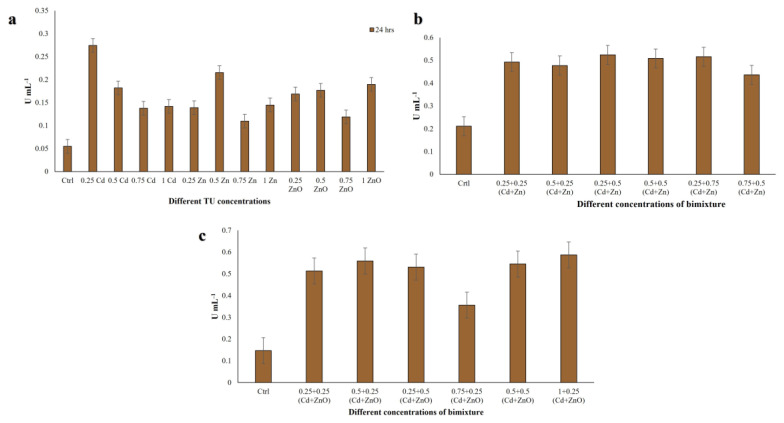
Superoxide dismutase (SOD) activity measured in *C. hirtus* cell extracts after 24 h exposure to: (**a**) individual HM (Cd, Zn) and NP (ZnO) treatments, (**b**) Cd + Zn mixtures and (**c**) Cd + ZnO mixtures. Results are presented as mean ± SE of three independent biological replicates.

SOD enzymes deal with the reactive oxygen species (ROS)/free radicals by consecutively adding or removing an electron from the superoxide molecules it encounters, thus changing the O_2_^−^ into one of two less damaging species: either molecular oxygen (O_2_) or hydrogen peroxide (H_2_O_2_). Superoxide is produced as a by-product of oxygen metabolism and, if not regulated, causes many types of cell damage [65]. Due to its importance in scavenging ROS and free radicals, we evaluated SOD activity in the *C. hirtus* extracts. SOD activity in single-compound exposures ranged from 0.11 to 0.27 U mL^−1^ (Figure 9a). The highest activity was observed at 0.25 TU Cd, whereas the lowest was recorded at 0.75 TU Zn. In Cd + Zn mixtures, SOD activity increased to values between 0.44 and 0.52 U mL^−1^ (Figure 9b), with the maximum at 0.25 + 0.5 TU and the minimum at 0.75 + 0.5 TU (Cd + Zn). In Cd + ZnO mixtures, SOD activity ranged from 0.36 to 0.59 U mL^−1^ (Figure 9c), with the lowest value at 0.75 + 0.25 TU and the highest at 1 + 0.25 TU (Cd + ZnO). Thus, SOD activity was enhanced under binary exposures compared to single metals, with the highest responses again associated with Cd + ZnO combinations. Data met the assumptions for parametric analysis (see Materials and Methods and Supplementary Tables S3 and S4).

### 3.5. Correlation Analyses Among Antioxidant Responses

#### 3.5.1. Correlations Among Non-Enzymatic Antioxidant Assays

Correlation coefficients (R) between TPC, DPPH, and HRSA are reported in Table 6. In single-compound treatments at 24 h, TPC vs. DPPH and TPC vs. HRSA showed strong positive correlations (R = 0.784 and R = 0.785, respectively), indicating that higher phenolic content was associated with higher radical scavenging capacity. DPPH vs. HRSA showed a moderate positive correlation (R = 0.665), reflecting that these assays, although related, respond to different radical species. In Cd + Zn mixtures at 24 h, TPC vs. DPPH remained moderately correlated (R = 0.666), while TPC vs. HRSA showed a strong correlation (R = 0.799). The DPPH vs. HRSA correlation increased markedly (R = 0.952), suggesting a highly coordinated antioxidant response under metal co-exposure. Overall, these patterns indicate that phenolic compounds are major contributors to antioxidant capacity in *C. hirtus*, and that their involvement becomes more tightly coupled with DPPH and HRSA under binary mixture stress.

#### 3.5.2. Correlations Among Antioxidant Enzymes

Correlation coefficients between antioxidant enzyme activities (CAT, GST, GPx, SOD) are summarized in Table 7. In single-compound treatments at 24 h, correlations were generally weak to moderate: CAT vs. GST (R = 0.364), CAT vs. GPx (R = 0.565), CAT vs. SOD (R = 0.490), GST vs. GPx (R = 0.5848), GST vs. SOD (R = 0.151), and GPx vs. SOD (R = 0.194). These values suggest that, under single-compound exposure, each enzyme responds somewhat independently, reflecting partially distinct roles in ROS detoxification. In contrast, under Cd + Zn co-exposure, all enzymes showed very strong positive correlations: CAT vs. GST (R = 0.945), CAT vs. GPx (R = 0.884), CAT vs. SOD (R = 0.952), GST vs. GPx (R = 0.938), GST vs. SOD (R = 0.992), and GPx vs. SOD (R = 0.947). This indicates that, in the presence of both metals, antioxidant enzymes respond in a highly coordinated and interdependent manner, forming an integrated defense system. Overall, correlation analyses reveal that *C. hirtus* mounts a more synchronized antioxidant response under binary metal stress than under single-metal exposure, consistent with the higher complexity and toxicity of metal mixtures.

## 4. Discussion

The present study provides the first integrated assessment of cytotoxicity, mixture toxicity, and antioxidant responses to HMs and metal-NPs in the freshwater ciliate *C. hirtus*. By combining acute lethality tests with biochemical biomarkers under both single and binary exposures, we expand current knowledge on ciliate stress physiology and evaluate the potential of this species as a bioindicator of metallic pollution.

### 4.1. Toxicity Profiles of HMs and NPs

Previous studies have shown that freshwater ciliates tend to be more sensitive to Cu than to other HMs such as Cd, Zn, Pb, Ni, and Cr [13]. Our results are consistent with this general pattern: Cu was the most toxic metal for *C. hirtus* (24 h LC_50_ = 1.62 mg L^−1^), followed by Cd (2.75 mg L^−1^) and Zn (20.42 mg L^−1^). This ranking agrees with observations in other ciliate species, including *Euplotes aediculatus*, *Euplotes crassus*, *Tetrahymena* spp., *Colpoda steinii*, and various soil ciliates [10,18,23,24,66,67,68], although the magnitude of LC_50_ values differs among taxa, reflecting species-specific tolerance. In our previous work, we characterized metal toxicity in *Rigidohymena tetracirrata* and *E. aediculatus* [10,66], two additional ciliates proposed as useful test organisms. When compared to those species, *C. hirtus* exhibits comparable or higher sensitivity to Cd and Zn, and a Cu sensitivity within the lower range of reported values. These features indicate that *C. hirtus* is neither unusually tolerant nor insensitive to these priority metals and can therefore detect ecologically relevant concentration ranges, which is desirable for bioindicator applications. NPs were substantially less toxic than their dissolved metal counterparts on a mass basis. ZnO and CuO exhibited intermediate toxicity, while TiO_2_ and especially SiO_2_ induced negligible mortality even at the highest tested concentrations. This pattern is in line with reports on other ciliates and microorganisms, where metal-oxide NPs frequently show lower acute toxicity than free metal ions and where toxicity is often linked to partial dissolution and ion release [20,21,22,67,69]. The very high LC_50_ values obtained for TiO_2_ and SiO_2_ are consistent with their low solubility and reactivity under the tested conditions. Overall, our data support the view that ionic forms of Cu, Cd, and Zn represent a greater acute hazard to *C. hirtus* than the NPs studied here.

### 4.2. Mixture Toxicity: Antagonism in Cd + Zn vs. Synergism in Cd + ZnO

The binary Cd + Zn mixture exhibited predominantly antagonistic interactions when evaluated using the TU approach (Table 2). In most combinations, observed mortality was lower than expected, indicating that the two metals partially reduce each other’s toxicity. This agrees with previous observations in fish, invertebrates, and microalgae, where Zn can mitigate Cd toxicity by competing for uptake sites or intracellular ligands [70,71,72,73,74]. MixTOX analysis corroborated this interpretation: the Independent Action (IA) model provided the best fit to the data (*R*^2^ = 0.93), and inclusion of IA-based deviation terms did not substantially improve model performance (Table 4), suggesting largely independent action with modest antagonistic deviations.

From a mechanistic perspective, the antagonistic behavior observed for Cd + Zn is consistent with competition between divalent metal ions for shared membrane transport systems and binding sites, which can reduce Cd internalization in the presence of elevated Zn. In addition, Zn exposure may induce metal-binding and detoxification responses, such as metallothionein-like sequestration or enhanced antioxidant capacity, thereby lowering the effective intracellular burden of Cd and attenuating its toxicity. Such antagonistic interactions between divalent metals are widely documented and commonly attributed to competitive uptake and detoxification mechanisms [62,75].

In contrast, the Cd + ZnO mixture displayed clear synergism over almost the entire TU range (Table 3). Here, basic CA and IA models alone did not adequately describe the data, whereas deviation models, especially IA/DR (dose-ratio-dependent deviation), significantly improved the fit (*R*^2^ up to 0.96; Table 5). The pronounced induction of both enzymatic and non-enzymatic antioxidant responses under Cd + ZnO exposure supports the interpretation that this mixture imposes a substantially higher oxidative burden than either component alone. A plausible explanation is that ZnO NPs contribute additional stress through particle-mediated interactions with cells and/or partial dissolution, releasing Zn^2+^, while Cd simultaneously disrupts redox homeostasis. The combined effects may overwhelm cellular defense systems, resulting in synergistic toxicity. Importantly, this interpretation is consistent with the observed dose-ratio-dependent synergism, suggesting threshold-like behavior in cellular stress responses rather than a simple additive effect. Similar nanoparticle-metal synergisms linked to oxidative stress and ion release have been reported in microbial and algal models [61,76,77]. This has important ecotoxicological implications, as natural waters are increasingly contaminated by both dissolved metals and engineered NPs.

### 4.3. Antioxidant Responses as Biomarkers of Oxidative Stress

HMs and NPs are known to induce ROS generation in microorganisms, leading to oxidative damage unless counteracted by antioxidant defenses [47,48,50,51,52,53,54,55,56,57,58]. In *C. hirtus*, we observed clear and statistically significant changes in both non-enzymatic and enzymatic antioxidant parameters following exposure to HMs and NPs, with particularly strong responses in binary mixtures. Non-enzymatic endpoints, including TPC, DPPH, and HRSA, were consistently elevated in exposed cells compared with controls, and often showed higher values in mixtures than in single-metal treatments, especially in Cd + ZnO. This indicates that phenolic-like compounds and other small-molecule antioxidants are mobilized as part of the defense strategy against ROS generated by metallic stress. Overall, this pattern supports the interpretation that low-molecular-weight antioxidant pools contribute substantially to the early cellular response to oxidative challenge in *C. hirtus*. The strong positive correlations between TPC and both DPPH and HRSA in single-metal exposures (R = 0.78) and the very high DPPH-HRSA correlation under Cd + Zn mixtures (R = 0.952; Table 6) suggest that these assays reflect closely related aspects of the non-enzymatic antioxidant system, which becomes more tightly coordinated as stress intensifies.

While antioxidant responses can be elicited by a variety of environmental stressors and are not unique to HMs or NPs, coordinated changes in multiple antioxidant biomarkers can provide mechanistic insight into toxic modes of action rather than serve as simple presence/absence signals. Accordingly, in this study, we treat antioxidant endpoints primarily as a mode-of-action readout that helps contextualize oxidative perturbation under single-compound and mixture exposures. Protists, including ciliates, occupy key positions in freshwater microbial food webs and respond rapidly to chemical stress, making them valuable early-response organisms [78]. Although antioxidant assays are more labor-intensive than lethality endpoints, they can detect sublethal oxidative perturbations and help interpret differences in physiological response patterns between single toxicants and mixtures, thereby enhancing ecological interpretation in complex exposure scenarios. Thus, the antioxidant profile of *C. hirtus* provides complementary physiological information beyond mortality-based endpoints.

Enzymatic antioxidants (CAT, GST, GPx, SOD) also showed pronounced, concentration-dependent activation. Activity levels were generally higher in binary mixtures than in single-compound exposures, with GST and SOD showing the strongest induction, particularly in Cd + Zn and Cd + ZnO treatments. This pattern points to an important role of glutathione-dependent detoxification and superoxide dismutation in the management of metal- and nanoparticle-induced oxidative stress in *C. hirtus*, consistent with observations in other protists and metazoans [52,79,80,81,82,83].

### 4.4. Mechanistic Interpretation of Antioxidant Responses Under Mixture Stress

The antagonistic interaction observed for the Cd + Zn mixture can be explained by the activation of protective detoxification and metal-sequestration mechanisms. Zn is known to induce metallothioneins and phase II detoxification enzymes, such as GST, which was markedly upregulated in this study. These responses promote intracellular binding and conjugation of Cd, thereby reducing its bioavailability and limiting Cd-driven oxidative damage. The coordinated increase in antioxidant enzymes (GST, CAT, SOD, and GPx) observed under Cd + Zn exposure is therefore consistent with a protective cellular state, in which Zn-mediated activation of detoxification and redox-balancing pathways attenuates Cd toxicity, resulting in an overall antagonistic interaction [62,75,84]. This interpretation is consistent with the strong, highly coordinated enzymatic correlation structure observed under Cd + Zn co-exposure (Table 7), suggesting an integrated antioxidant network that may contribute to buffering oxidative injury at specific Cd:Zn ratios.

In contrast, the strong synergism observed for the Cd + ZnO mixture is supported by the pronounced and coordinated induction of both enzymatic and non-enzymatic antioxidant defenses, indicating severe oxidative stress that exceeds cellular defense capacity. ZnO NPs can contribute to toxicity through particle-mediated ROS generation and, potentially, partial dissolution with Zn^2+^ release, while Cd disrupts redox homeostasis and interferes with antioxidant regulation. When combined, the cumulative oxidative burden may overwhelm detoxification and repair systems, consistent with the markedly elevated antioxidant responses observed in this mixture. Such oxidative overload may also contribute to secondary effects (e.g., membrane destabilisation), thereby increasing susceptibility to uptake and intracellular toxicity. In addition, nanoparticle–metal interactions, such as adsorption of Cd onto ZnO surfaces or nanoparticle-mediated co-transport, may further increase intracellular Cd delivery, amplifying toxicity. The marked elevation of GST activity supports the activation of conjugation-based detoxification pathways in response to excessive oxidative and electrophilic stress, while the dose-ratio-dependent synergism detected by MixTOX suggests threshold effects in cellular defense mechanisms, whereby increasing combined stress results in non-linear toxicity amplification [61,62,76,77,85,86].

Beyond the magnitude of individual responses, the correlation structure among antioxidant enzymes provides insight into how *C. hirtus* organizes its defense system under different stress scenarios. In single-compound treatments, correlations among CAT, GST, GPx, and SOD were weak to moderate (R ≤ 0.58; Table 7), suggesting that these enzymes are regulated in a relatively independent fashion and may respond to distinct ROS signals or damage types.

In contrast, under Cd + Zn co-exposure, all enzyme pairs displayed very strong positive correlations (R ≥ 0.884), with GST-SOD approaching unity (R = 0.992). This indicates a shift toward a highly coordinated enzymatic response when the organism faces a more complex stressor, such as a metal mixture. Such coordination implies that the antioxidant system operates as an integrated network rather than a set of isolated pathways, and that multiple enzymes could serve as mutually reinforcing biomarkers in mixture toxicity assessments. For Cd + ZnO, the strong induction of multiple antioxidant endpoints likewise supports an integrated response under synergistic stress, even though correlation coefficients for that mixture are not reported here.

### 4.5. Ecological Rationale and Bioindicator Potential of Coleps hirtus

The bioindicator potential of *C. hirtus* is strongly supported by its carnivorous and scavenging lifestyle, which exposes it to contaminants through both the water column and ingestion of contaminated prey, thereby integrating multiple exposure pathways. This dual route of exposure may enhance sensitivity to complex contaminant mixtures and increase the likelihood of detecting biologically relevant interactions that may be underestimated in primary producers or single-endpoint assays. As ciliates occupy a key trophic position linking microbial producers to higher consumers, contaminant effects in *C. hirtus* may propagate through freshwater food webs, increasing ecological relevance [87,88,89]. In this context, the strong response of *C. hirtus* to the Cd + ZnO combination observed in the present study highlights its potential as an early-warning organism in mixed-contamination settings. Although ciliates such as *Tetrahymena* and *Euplotes* are widely used in ecotoxicological studies due to their ease of culture and standardized protocols, *C. hirtus* offers complementary advantages linked to its distinct ecological role and feeding strategy. *Tetrahymena* is primarily bacterivorous and commonly used as a laboratory model organism, while *Euplotes* is largely substrate-associated and feeds on bacteria and microalgae. In contrast, *C. hirtus* is a free-swimming, carnivorous and scavenging ciliate, preying on other protists and detrital material, increasing the likelihood of contaminant uptake from both the water column and particulate-associated food sources, including sediment-associated particles. By integrating multiple exposure routes that more closely resemble natural conditions, *C. hirtus* can complement established ciliate models rather than replace them, broadening ecotoxicological assessment to trophic strategies and exposure pathways that are underrepresented in classical laboratory systems [61,87,88].

### 4.6. Ecotoxicological Relevance of C. hirtus and Implications for Biomonitoring

Overall, the patterns observed in this study support *C. hirtus* as a promising bioindicator for freshwater environments impacted by HMs and metal-oxide NPs. Its clear sensitivity to Cu, Cd, and Zn, together with measurable responses to ZnO and CuO, demonstrates that this ciliate can detect contamination across a broad range of metallic forms while remaining robust and easy to handle under laboratory conditions. A key strength of *C. hirtus* lies in its coherent biomarker profile: both non-enzymatic and enzymatic antioxidant parameters respond in a dose-dependent fashion and become strongly coordinated under mixture exposure, providing a sensitive and mechanistically informative readout of oxidative stress. Importantly, the mixture experiments revealed contrasting interaction outcomes depending on contaminant composition, with predominantly antagonistic effects observed in Cd + Zn and pronounced synergistic toxicity in Cd + ZnO. These distinct mixture-dependent response patterns are particularly relevant for modern ecotoxicology, where risk is increasingly shaped by the co-occurrence of multiple contaminants rather than by single substances in isolation. Taken together, these characteristics suggest that *C. hirtus* could be integrated into ecotoxicological test batteries to support hazard screening and mixture-focused assessments in freshwater systems. Given the increasing likelihood of co-occurrence of dissolved metals and engineered NPs in impacted waters (e.g., industrial and municipal effluents), *C. hirtus* may be especially informative in monitoring contexts where mixture interactions are expected to influence risk. Future work should prioritize longer exposures, additional endpoints (e.g., reproduction, feeding or behavior), and more complex mixtures to refine its applicability and operational use in biomonitoring frameworks.

## 5. Limitations and Future Perspectives

The present study provides a comprehensive assessment of the acute toxicity, mixture interactions, and antioxidant responses of *C. hirtus* exposed to HMs and metal-oxide NPs. Nevertheless, several limitations should be acknowledged in order to appropriately frame the interpretation of the results and to guide future investigations. A primary limitation concerns the characterization of NPs’ behavior in the exposure medium. Although nominal particle size and BET surface area were reported based on supplier specifications, quantitative medium physicochemical characterization, such as hydrodynamic size distribution, aggregation kinetics, surface charge, and dissolved metal release, was not performed. As recognized by OECD TG 318 [49], NP dispersion stability in aqueous media is dynamic, time-dependent, and strongly influenced by medium composition. In the present study, NP suspensions were freshly prepared for each experiment and dispersion stability was qualitatively monitored between 0 and 24 h by visual inspection for sedimentation or aggregation. However, future studies should integrate OECD TG 318 [49] based quantitative dispersion stability testing and dissolved metal measurements (e.g., Zn^2+^ release from ZnO) to refine exposure characterization and to better disentangle particulate versus ionic contributions to toxicity. A second limitation relates to the mechanistic resolution of the observed mixture effects. While the combined use of Toxic Unit analysis, MixTOX modeling, and coordinated antioxidant biomarkers provides robust evidence for antagonistic interactions in Cd + Zn mixtures and synergistic interactions in Cd + ZnO mixtures, direct molecular or cellular targets were not investigated. Future work should therefore incorporate complementary endpoints, such as intracellular metal accumulation, oxidative damage markers, membrane integrity assays, and omics-based approaches, to further elucidate the mechanisms underlying metal-NP interactions. Finally, the present experiments were conducted under controlled laboratory conditions using acute exposure scenarios. Although this approach is appropriate for comparative toxicity assessment and mixture interaction analysis, it does not fully capture the complexity of natural freshwater systems. Future research should explore chronic and sub-chronic exposures, environmentally realistic concentrations, dietary exposure routes, and complex effluent matrices to strengthen the ecological relevance of *C. hirtus* based assessments. Addressing these limitations will further improve the interpretative power and applicability of *C. hirtus* in freshwater ecotoxicology, particularly for evaluating risks associated with emerging contaminants and complex chemical mixtures.

## 6. Conclusions

This study provides the first quantitative assessment of the ecotoxicological responses of *C. hirtus* to HMs and metal-oxide NPs by integrating acute toxicity, mixture analysis, and antioxidant biomarkers. The results demonstrate that *C. hirtus* exhibits distinct sensitivity patterns across contaminants, clearly differentiating between the effects of dissolved metal and nanoparticulate forms. Mixture experiments showed that contaminant speciation strongly influences interaction outcomes, with predominantly antagonistic responses characterizing Cd + Zn mixtures and pronounced synergism observed in Cd + ZnO. These contrasting patterns highlight the importance of explicitly considering mixture composition and chemical form in ecotoxicological assessments, particularly in aquatic environments where traditional metal pollutants increasingly co-occur with engineered nanomaterials.

At sub-lethal levels, *C. hirtus* displayed coordinated modulation of enzymatic and non-enzymatic antioxidant defenses consistent with oxidative stress, supporting the use of physiological biomarkers to complement mortality-based endpoints. Taken together, the sensitivity of *C. hirtus* to both single contaminants and mixtures, combined with its ecological relevance, supports its suitability as a promising freshwater bioindicator for assessing complex metallic and nanomaterial contamination.

## Figures and Tables

**Table 1:** 24 h LC_20_ and LC_50_ values for HMs and NPs in *C. hirtus* from logit model analysis.

S. No:		Parameter	Estimate (± SE)	95% Confidence Interval	*R* ^2^
		**Heavy Metals**			
1	Cu	LC_20_	0.87 ± 0.05	0.77–0.97	0.997
		LC_50_	1.62 ± 0.04	1.55–1.69	
2	Zn	LC_20_	8.26 ± 0.68	6.93–9.59	0.987
		LC_50_	20.42 ± 0.89	18.68–22.16	
3	Cd	LC_20_	1.47 ± 0.08	1.32–1.62	0.994
		LC_50_	2.75 ± 0.06	2.63–2.87	
		**Nanoparticles**			
1	CuO	LC_20_	256.45 ± 15.32	226.43–286.47	0.996
		LC_50_	447.83 ± 11.94	424.43–471.23	
2	ZnO	LC_20_	138.72 ± 9.85	119.41–158.03	0.993
		LC_50_	356.18 ± 16.32	324.19–388.17	
3	TiO_2_	LC_20_	6487.15 ± 462.25	5581.14–7393.16	0.995
		LC_50_	12,879.33 ± 405.67	12,084.22–13,679.44	
4	SiO_2_	LC_20_	Up to 60,080 mg L^−1^, there are no effects on the cells		
		LC_50_			

Note: concentrations are in mg L^−1^.

**Table 2:** Observed and predicted cytotoxicity (% mortality) of *C. hirtus* to different mixtures of CdCl_2_ + ZnSO_4_ after 24 h exposition.

Total TU ^a^	CdCl_2_ TU ^a^	ZnSO_4_ TU ^a^	Obtained Cytotoxicity ^b^	Expected Cytotoxicity ^b^	Interaction Type
0.5	0.25	0.25	13.00 ± 3.04	15 ± 2.3	Not significant different
0.75	0.5	0.25	27.11 ± 1.62	29 ± 2.7	Not significant different
	0.25	0.5	20.89 ± 1.27	26 ± 2.7	Antagonism
1	0.75	0.25	49.56 ± 2.60	46 ± 2.8	Synergism
	0.5	0.5	34.00 ± 1.22	40 ± 2.8	Antagonism
	0.25	0.75	35.44 ± 3.28	38 ± 2.8	Not significant different
1.25	0.75	0.5	42.56 ± 1.33	57 ± 2.6	Antagonism
	0.5	0.75	56.67 ± 2.60	52 ± 2.6	Synergism
	1	0.25	55.00 ± 2.80	62 ± 2.5	Antagonism
	0.25	1	66.89 ± 2.52	53 ± 2.6	Synergism
1.5	0.5	1	72.67 ± 2.35	67 ± 2.3	Synergism
	0.75	0.75	53.00 ± 1.00	69 ± 2.3	Antagonism
	1	0.5	60.33 ± 1.58	73 ± 2.2	Antagonism
1.75	1	075	81.00 ± 2.12	85 ± 1.7	Antagonism
	0.75	1	87.00 ± 2.74	84 ± 1.7	Not significant different
2	1	1	96.56 ± 2.60	100 ± 0.3	Not significant different

^a^ Toxic Units, mg L^−1^. ^b^ % mortality ± standard deviation. Footnotes: TU = 1 corresponds to the LC_50_ of each individual toxicant; Interaction outcomes were classified by comparing observed and expected cytotoxicity values: synergistic (observed > expected), antagonistic (observed < expected), and additive (no significant difference between observed and expected values).

**Table 3:** Observed and predicted cytotoxicity (% mortality) of *C. hirtus* to different mixtures of CdCl_2_ + ZnO after 24 h exposition.

Total TU ^a^	CdCl_2_ TU ^a^	ZnO TU ^a^	Obtained Cytotoxicity ^b^	Expected Cytotoxicity ^b^	Interaction Type
0.5	0.25	0.25	42.33 ± 1.94	8 ± 0.3	Synergism
0.75	0.5	0.25	51.67 ± 1.87	22 ± 1.0	Synergism
	0.25	0.5	74.11 ± 1.45	22 ± 1.0	Synergism
1	0.75	0.25	67.44 ± 2.46	39 ± 2.1	Synergism
	0.5	0.5	84.56 ± 1.42	36 ± 2.0	Synergism
	0.25	0.75	100.00 ± 0.00	36 ± 2.0	Synergism
1.25	0.75	0.5	88.67 ± 1.00	53 ± 2.4	Synergism
	0.5	0.75	100.00 ± 0.00	49 ± 2.4	Synergism
	1	0.25	76.22 ± 2.11	55 ± 2.3	Synergism
	0.25	1	100.00 ± 0.00	53 ± 2.4	Synergism
1.5	0.5	1	100.00 ± 0.00	67 ± 2.0	Synergism
	0.75	0.75	100.00 ± 0.00	66 ± 2.0	Synergism
	1	0.5	95.11 ± 1.27	69 ± 1.9	Synergism
1.75	1	075	100.00 ± 0.00	82 ± 1.3	Synergism
	0.75	1	100.00 ± 0.00	84 ± 1.2	Synergism
2	1	1	100.00 ± 0.00	100 ± 0.2	Not significant different

^a^ Toxic Units, mg L^−1^. ^b^ % mortality ± standard deviation. Footnotes: TU = 1 corresponds to the LC_50_ of each individual toxicant; Interaction outcomes were classified by comparing observed and expected cytotoxicity values: synergistic (observed > expected), antagonistic (observed < expected), and additive (no significant difference between observed and expected values).

**Table 4:** Fitting results of the toxicity of the binary mixture (Cd + Zn) data to the four models describing deviations from concentration addition (CA) and independent action (IA) of *C. hirtus* by the MixTOX tool.

*C. hirtus* CdCl_2_ + ZnSO_4_	Parameter	The Concentration Addition (CA) Based Module			
Exposures		CA	S/A	DR	DL
24 h	*a*	–	0.487	0.087	1.829
	*b*	–	–	0.783	0.583
	*R* ^2^	0.88	0.90	0.90	0.91
	*p* (*χ*^2^) CA vs.	–	6.45 × 10^−7^ *	2.04 × 10^−6^ *	4.71 × 10^−9^ *
	S/A vs.	–	–	0.231	0.0002 *
	**Parameter**	**The Independent action (IA) based module**			
		**IA**	**S/A**	**DR**	**DL**
24 h	*a*	–	−0.147	−0.689	−0.047
	*b*	–	–	1.108	−3.925
	*R* ^2^	0.93	0.93	0.91	0.93
	*p* (*χ*^2^) IA vs.	–	0.331	0.343	0.604
	S/A vs.	–	–	0.274	0.802

24 h: 24 h exposure period; CA: the concentration addition model; IA: the independent addition model; *a* and *b*: interaction parameters; S/A: the model describing synergy or antagonism in relation to the reference model has one interaction parameter (*a*); DR: the model describing dose ratio-dependent deviations from the reference model has two interaction parameters (*a* and *b*); DL: the model describing dose level-dependent deviations from the reference model has two interaction parameters (*a* and *b*); *R*^2^: the coefficient of determination; *p* (*χ*^2^): the outcome of the likelihood ratio test; vs.: versus, which was used to show comparison between two models; –: not applicable. *: Significant at the 5% significant level.

**Table 5:** Fitting results of the toxicity of the binary mixture (Cd + ZnO) data to the four models describing deviations from concentration addition (CA) and independent action (IA) of *C. hirtus* by the MixTOX tool.

*C. hirtus* CdCl_2_ + ZnO	Parameter	The Concentration Addition (CA) Based Module			
Exposures		CA	S/A	DR	DL
24 h	*a*	–	−1.986	−5.702	−0.820
	*b*	–	–	7.118	−1.705
	*R* ^2^	0.77	0.93	0.96	0.93
	*p* (*χ*^2^) CA vs.	–	2.2 × 10^−80^ *	5.5 × 10^−100^ *	7.99 × 10^−81^ *
	S/A vs.	–	–	8.36 × 10^−23^ *	0.003 *
	**Parameter**	**The Independent action (IA) based module**			
		**IA**	**S/A**	**DR**	**DL**
24 h	*a*	–	−5.629	−12.304	−7.321
	*b*	–	–	12.741	0.008
	*R* ^2^	0.33	0.92	0.96	0.90
	*p* (*χ*^2^) IA vs.	–	0.000 *	0.000 *	0.000 *
	S/A vs.	–	–	7.75 × 10^−22^ *	1.03 × 10^−6^ *

24 h: 24 h exposure period; CA: the concentration addition model; IA: the independent addition model; *a* and *b*: interaction parameters; S/A: the model describing synergy or antagonism in relation to the reference model has one interaction parameter (*a*); DR: the model describing dose ratio-dependent deviations from the reference model has two interaction parameters (*a* and *b*); DL: the model describing dose level-dependent deviations from the reference model has two interaction parameters (*a* and *b*); *R*^2^: the coefficient of determination; *p* (*χ*^2^): the outcome of the likelihood ratio test; vs.: versus, which was used to show comparison between two models; –: not applicable. *: Significant at the 5% significant level.

**Table 6:** Correlation Coefficient (*R*) between single and binary mixture compound exposures of different antioxidant assays on *C. hirtus*.

	Single-Compound Treatment 24 h		
	TPC	DPPH	HRSA
**TPC**	1		
**DPPH**	0.784	1	
**HRSA**	0.785	0.665	1
	**Binary mixture (Cd + Zn) 24 h**		
	**TPC**	**DPPH**	**HRSA**
**TPC**	1		
**DPPH**	0.666	1	
**HRSA**	0.799	0.952	1

**Table 7:** Correlation Coefficient (*R*) between single and binary mixture exposures of different antioxidant enzyme assays on *C. hirtus*.

	Single-Compound Treatment 24 h			
	CAT	GST	GPx	SOD
**CAT**	1			
**GST**	0.364	1		
**GPx**	0.565	0.5848	1	
**SOD**	0.490	0.151	0.194	1
	**Binary mixture (Cd + Zn) 24 h**			
	**CAT**	**GST**	**GPx**	**SOD**
**CAT**	1			
**GST**	0.945	1		
**GPx**	0.884	0.938	1	
**SOD**	0.952	0.992	0.947	1

## Data Availability

The original contributions presented in this study are included in the article/Supplementary Material. Further inquiries can be directed to the corresponding author.

## Supplementary Materials

The following supporting information can be downloaded at: https://www.mdpi.com/article/10.3390/jox16010023/s1. Figure S1: Concentration-response relationships for *C. hirtus* after 24 h exposure to single compounds, used for the calculation of expected mortality in the Toxic Unit (TU) approach. Curves are shown for (**a**) Cd, (**b**) Zn, and (**c**) ZnO, with concentrations expressed in mg L^−1^. Table S1. Shapiro–Wilk normality test results for non-enzymatic antioxidant endpoints (TPC, DPPH, HRSA) in *C. hirtus* after 24 h exposure to single compounds, Cd + ZnSO_4_ mixtures, and Cd + ZnO mixtures. Table S2. Levene’s test results for homogeneity of variances for non-enzymatic antioxidant endpoints (TPC, DPPH, HRSA) in *C. hirtus* after 24 h exposure to single compounds, Cd + ZnSO_4_ mixtures, and Cd + ZnO mixtures. Table S3. Shapiro–Wilk normality test results for non-enzymatic antioxidant endpoints (CAT, GST, GPx, SOD) in *C. hirtus* after 24 h exposure to single compounds, Cd + ZnSO_4_ mixtures, and Cd + ZnO mixtures. Table S4. Levene’s test results for homogeneity of variances for non-enzymatic antioxidant endpoints (CAT, GST, GPx, SOD) in *C. hirtus* after 24 h exposure to single compounds, Cd + ZnSO_4_ mixtures, and Cd + ZnO mixtures. File S1: Original images of Figure 1.
