# Supplementary material for: The Freshwater Ciliate Coleps hirtus as a Model Organism for Metal and Nanoparticle Toxicity: Mixture Interactions and Antioxidant Responses
Source: J Xenobiot. 2026 Feb 1;16(1):23. doi: 10.3390/jox16010023 (PMC12922124; doi:10.3390/jox16010023)
Supplement: Supplementary file 1 [file jox-16-00023-s001.zip › jox-4080865-FileS1-Original images of Figure 1.pdf]

Journal of
Xenobiotics

MDPI

Supplementary Materials: The Freshwater Ciliate *Coleps hirtus* as a Model Organism for Metal and Nanoparticle Toxicity: Mixture Interactions and Antioxidant Responses

Govindhasamay R Varatharajan, Martina Coletta, Santosh Kumar, Daizy Bharti, Arnab Ghosh, Shikha Singh, Amit C. Kharkwal, Francesco Dondero and Antonietta La Terza

Original Images:

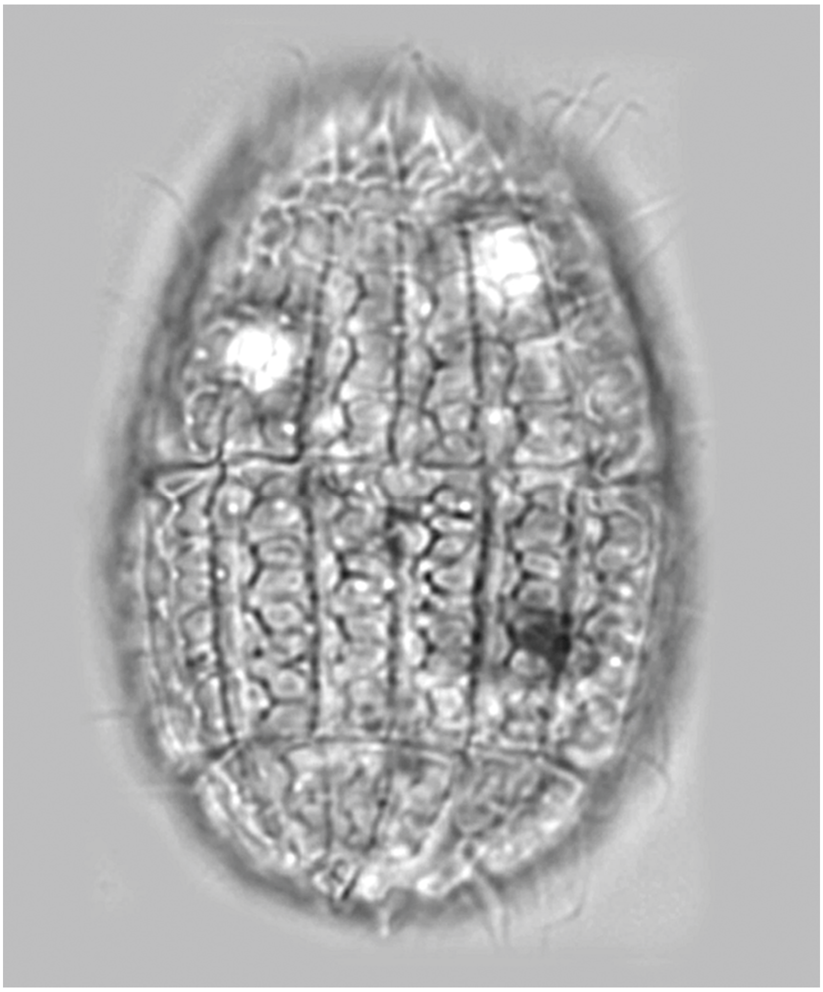

Figure 1A.

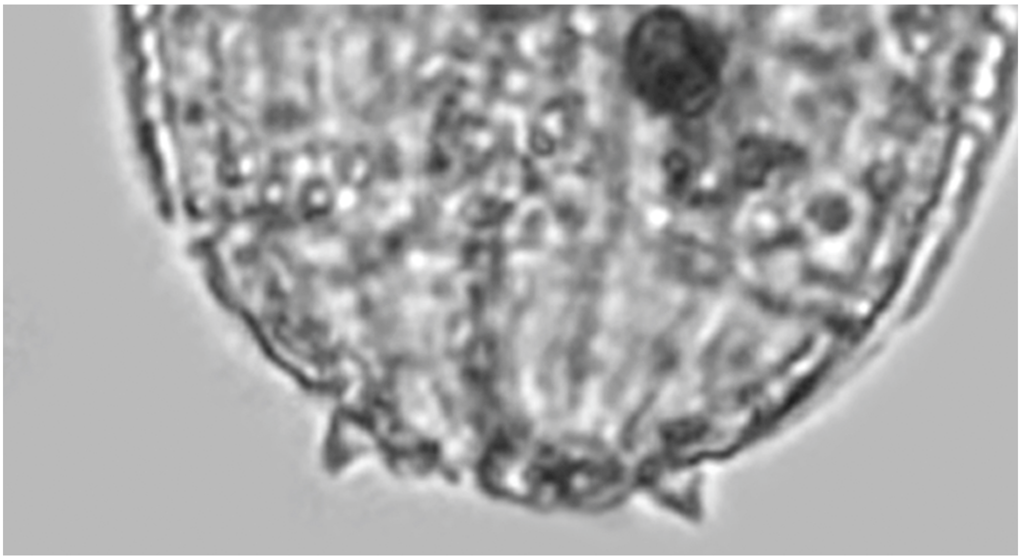

Figure 1B.

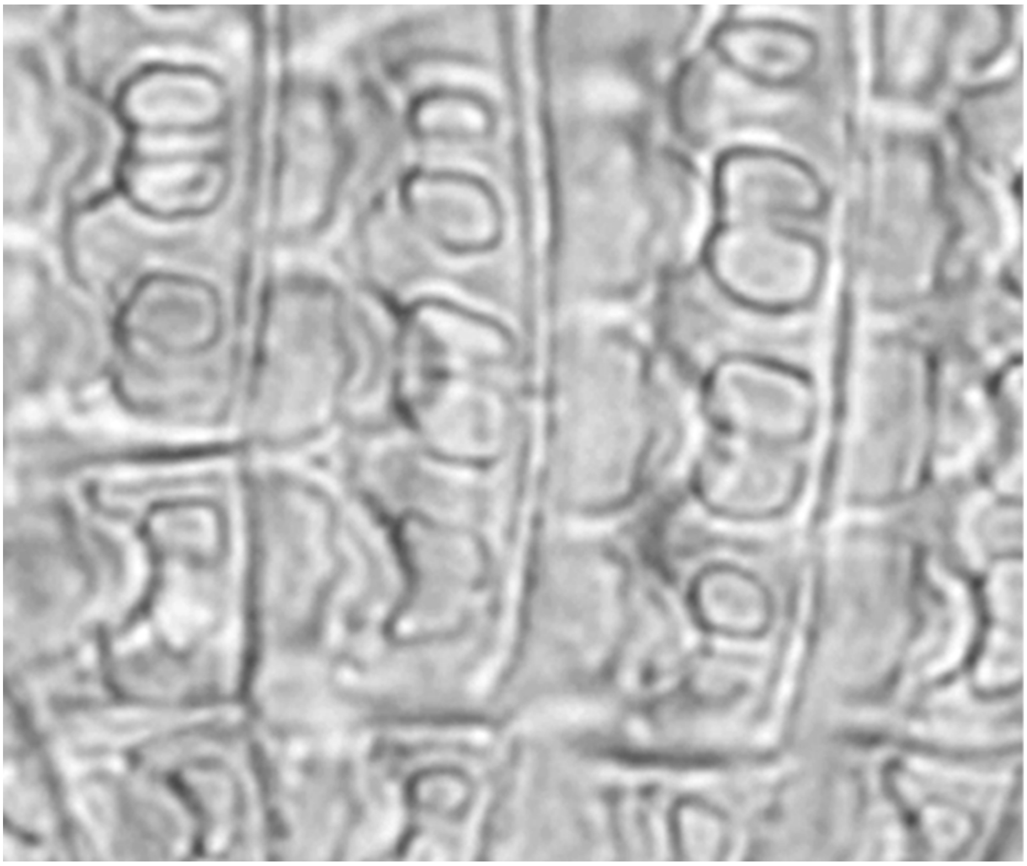

Figure 1C.

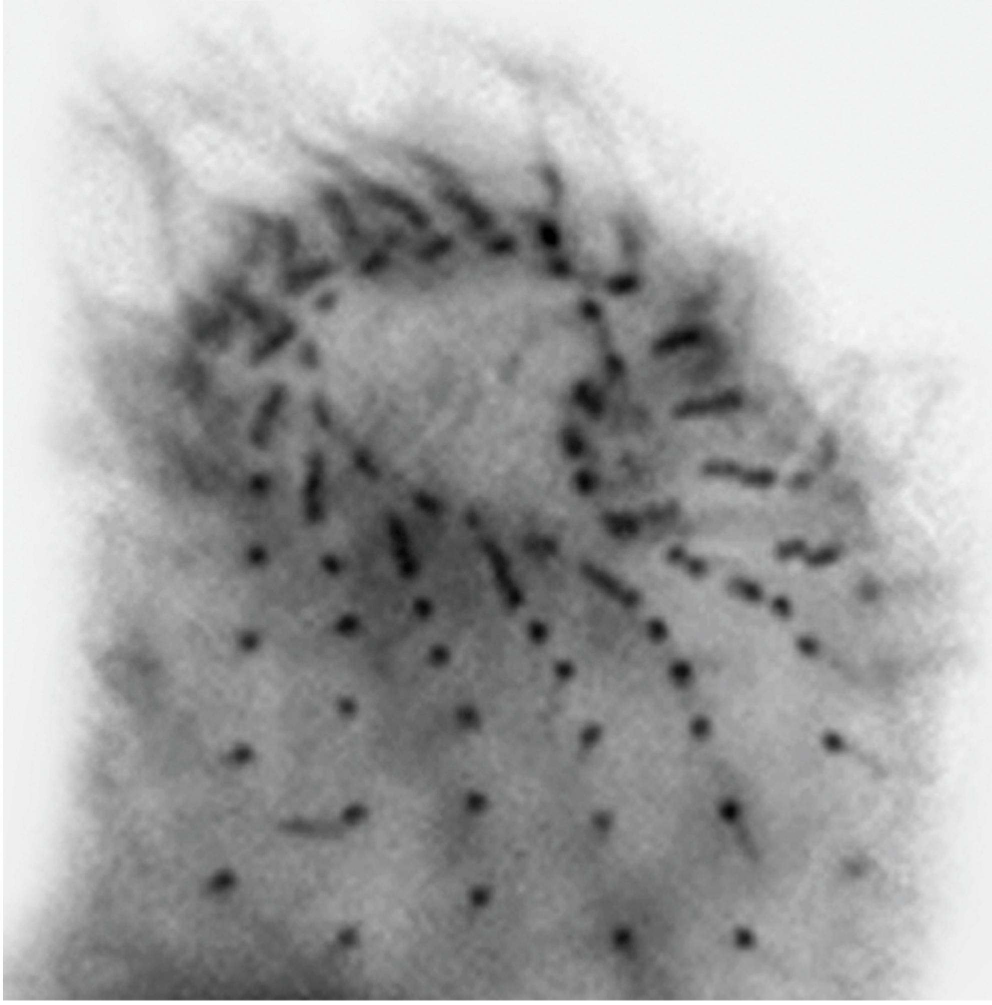

Figure 1D.

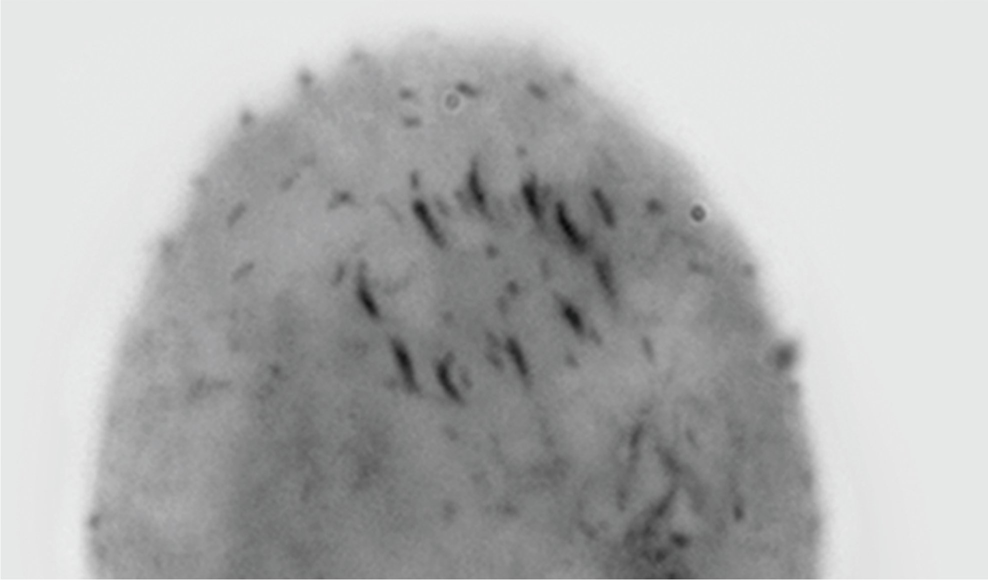

Figure 1E.

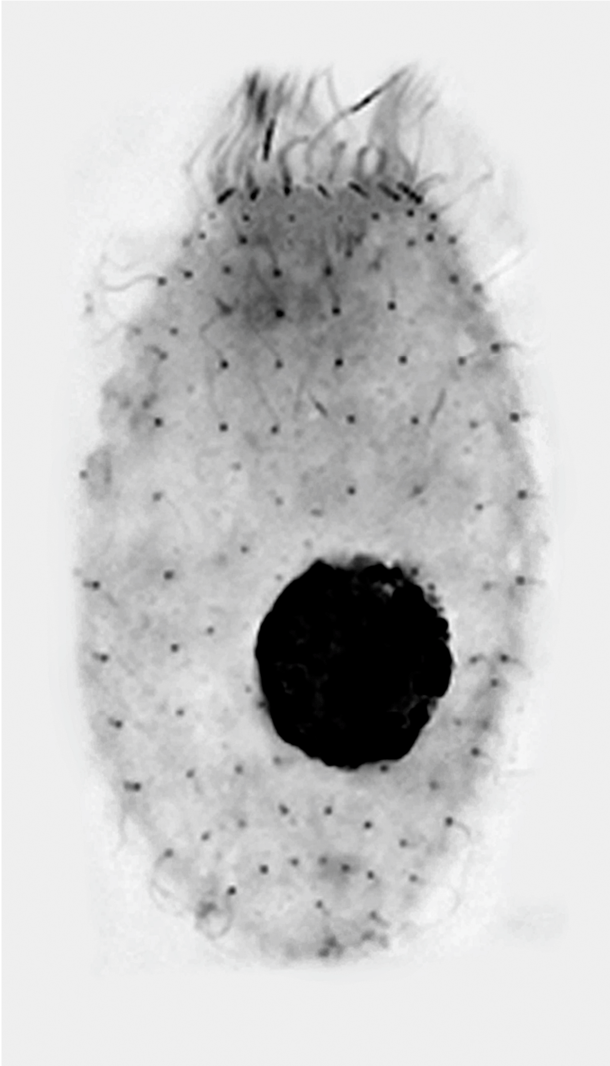

Figure 1F
