# Supplementary material for: The Freshwater Ciliate Coleps hirtus as a Model Organism for Metal and Nanoparticle Toxicity: Mixture Interactions and Antioxidant Responses
Source: J Xenobiot. 2026 Feb 1;16(1):23. doi: 10.3390/jox16010023 (PMC12922124; doi:10.3390/jox16010023)
Supplement: Supplementary file 1 [file jox-16-00023-s001.zip › jox-4080865-supplementary-proof.pdf]

Supplementary Materials: The Freshwater *Ciliate Coleps hirtus* as a Model Organism for Metal and Nanoparticle Toxicity: Mixture Interactions and Antioxidant Responses

Govindhasamay R. Varatharajan, Martina Coletta, Santosh Kumar, Daizy Bharti, Arnab Ghosh, Shikha Singh, Amit C. Kharkwal, Francesco Dondero and Antonietta La Terza

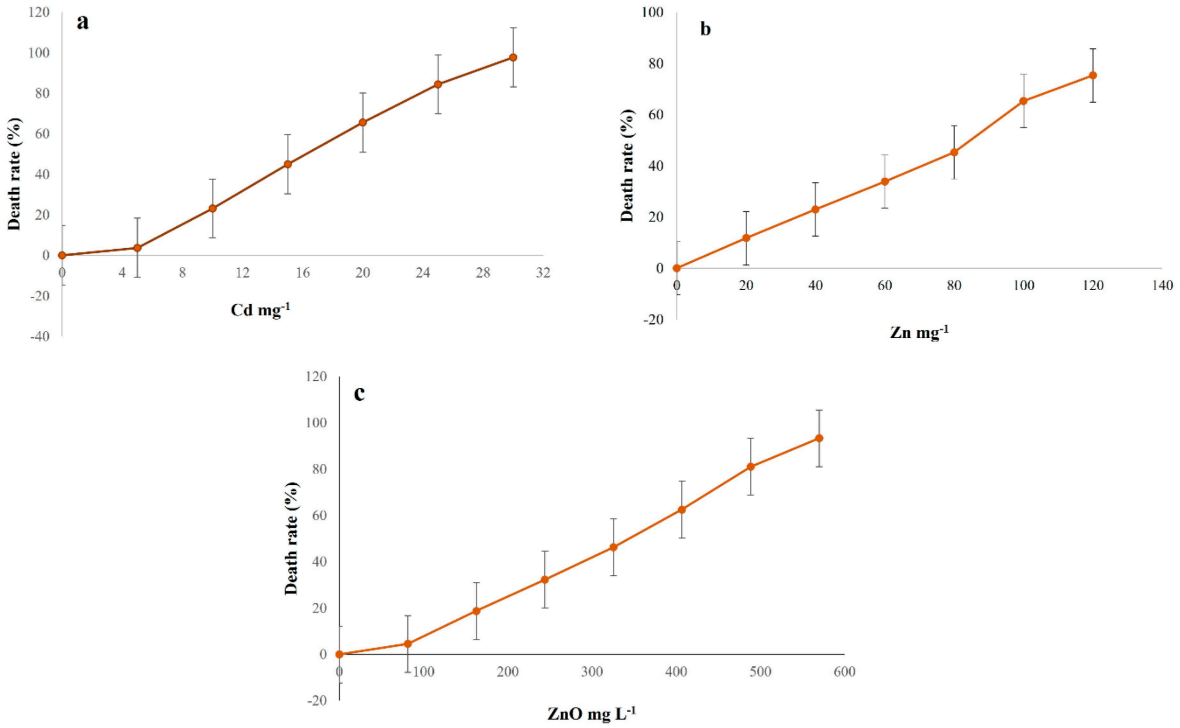

Figure S1. Concentration-response relationships for *C. hirtus* after 24 h exposure to single compounds, used for the calculation of expected mortality in the Toxic Unit (TU) approach. Curves are shown for (a) Cd, (b) Zn, and (c) ZnO, with concentrations expressed in mg L^{-1}.

Table S1. Shapiro–Wilk normality test results for non-enzymatic antioxidant endpoints (TPC, DPPH, HRSA) in *C. hirtus* after 24 h exposure to single compounds, Cd + $ZnSO_4$ mixtures, and Cd + ZnO mixtures.

Assay	Experiment	No. of groups tested	Groups with p > 0.05	Normality satisfied
TPC	Single	12	12	Yes
TPC	HM_Mix (Cd+$ZnSO_4$)	6	6	Yes
TPC	NP_Mix (Cd+ZnO)	6	6	Yes
DPPH	Single	5	5	Yes
DPPH	HM_Mix (Cd+$ZnSO_4$)	6	6	Yes
DPPH	NP_Mix (Cd+ZnO)	6	6	Yes
HRSA	Single	5	5	Yes
HRSA	HM_Mix (Cd+$ZnSO_4$)	6	6	Yes
HRSA	NP_Mix (Cd+ZnO)	6	6	Yes

p-values > 0.05 indicate no significant deviation from normality.

Table S2. Levene's test results for homogeneity of variances for non-enzymatic antioxidant endpoints (TPC, DPPH, HRSA) in *C. hirtus* after 24 h exposure to single compounds, Cd + $ZnSO_4$ mixtures, and Cd + ZnO mixtures.

Assay	Experiment	Levene's value	p- Homogeneity satisfied
TPC	Single	0.98	Yes
TPC	Cd+$ZnSO_4$	0.63	Yes
TPC	Cd+ZnO	0.76	Yes
DPPH	Single	> 0.60	Yes
DPPH	Cd+$ZnSO_4$	> 0.70	Yes
DPPH	Cd+ZnO	> 0.65	Yes
HRSA	Single	> 0.80	Yes
HRSA	Cd+$ZnSO_4$	> 0.70	Yes
HRSA	Cd+ZnO	0.76	Yes

p-values > 0.05 indicate no significant deviation from homogeneity of variances.

Table S3. Shapiro–Wilk normality test results for non-enzymatic antioxidant endpoints (CAT, GST, GPx, SOD) in *C. hirtus* after 24 h exposure to single compounds, Cd + $ZnSO_4$ mixtures, and Cd + ZnO mixtures.

Assay	Experiment	No. of groups tested	Groups with p > 0.05	Normality satisfied
CAT	Single	13	12	Yes
CAT	HM_Mix (Cd+$ZnSO_4$)	7	7	Yes
CAT	NP_Mix (Cd+ZnO)	7	6	Yes
GST	Single	13	12	Yes
GST	HM_Mix (Cd+$ZnSO_4$)	7	7	Yes
GST	NP_Mix (Cd+ZnO)	7	6	Yes
GPx	Single	13	11	Yes
GPx	HM_Mix (Cd+$ZnSO_4$)	7	7	Yes
GPx	NP_Mix (Cd+ZnO)	7	6	Yes
SOD	Single	13	11	Yes
SOD	HM_Mix (Cd+$ZnSO_4$)	7	7	Yes
SOD	NP_Mix (Cd+ZnO)	7	5	Yes

p-values > 0.05 indicate no significant deviation from normality.

Table S4. Levene's test results for homogeneity of variances for non-enzymatic antioxidant endpoints (CAT, GST, GPx, SOD) in *C. hirtus* after 24 h exposure to single compounds, Cd + $ZnSO_4$ mixtures, and Cd + ZnO mixtures.

Assay	Experiment	Levene's p-value	Homogeneity satisfied
CAT	Single	0.57	Yes
CAT	HM_Mix (Cd+$ZnSO_4$)	0.93	Yes
CAT	NP_Mix (Cd+ZnO)	0.90	Yes
GST	Single	0.95	Yes
GST	HM_Mix (Cd+$ZnSO_4$)	0.99	Yes
GST	NP_Mix (Cd+ZnO)	0.46	Yes
GPx	Single	0.48	Yes
GPx	HM_Mix (Cd+$ZnSO_4$)	0.72	Yes
GPx	NP_Mix (Cd+ZnO)	0.56	Yes
SOD	Single	0.89	Yes
SOD	HM_Mix (Cd+$ZnSO_4$)	0.67	Yes
SOD	NP_Mix (Cd+ZnO)	0.81	Yes

p-values > 0.05 indicate no significant deviation from homogeneity of variances.
